# Clearing the Air to Address Pollution’s Cardiovascular Health Crisis

**DOI:** 10.5334/gh.1364

**Published:** 2024-10-30

**Authors:** Mark R. Miller, Mariachiara Di Cesare, Shadi Rahimzadeh, Marvellous Adeoye, Pablo Perel, Sean Taylor, Shreya Shrikhande, Kelcey Armstrong-Walenczak, Anoop S. V. Shah, César Damián Berenstein, Rajesh Vedanthan, Elvis Ndikum Achiri, Sumi Mehta, Abiodun Moshood Adeoye, Daniel PiÑeiro, Fausto J. Pinto

**Affiliations:** 1 Centre for Cardiovascular Science, University of Edinburgh, UK; 2 Institute of Public Health and Wellbeing, University of Essex, Colchester, UK; 3 Department of Non-Communicable Disease Epidemiology, London School of Hygiene & Tropical Medicine, UK; 4 World Heart Federation, Geneva, Switzerland; 5 Director of ‘Cardioecology and Healthy Habits’Council, Argentine Society of Cardiology, AR; 6 Department of Population Health Institute for Excellence in Health Equity NYU Grossman School of Medicine New York, USA; 7 Institute of Medical Epidemiology, Biometry and Informatics, Martin Luther University, Halle Saale, Germany; 8 Vital Strategies, New York, USA; 9 Department of Medicine, University College Hospital, Ibadan, Nigeria; 10 Department of Medicine, University of Buenos Aires, Buenos Aires, Argentina; 11 Santa Maria University Hospital, CAML, CCUL, Faculdade de Medicina da Universidade de Lisboa, Lisbon, Portugal

**Keywords:** Ambient air pollution, Cardiovascular health, CVD global data, World Heart Observatory

## Abstract

Air pollution is a critical global health issue that significantly impacts cardiovascular health. The air pollutant PM_2.5_ (particulate matter with a diameter of 2.5 micrometres or less) has been positioned as a leading environmental risk factor for morbidity and mortality, especially from cardiovascular diseases (CVDs).

Using data from the World Health Organization (WHO), Global Health Observatory, and the United Nations Environment Programme, we explored global trends in air pollution, with a focus on PM_2.5_ levels, the implications for cardiovascular health, and the policy measures aimed at reducing their impact.

Despite progress in reducing pollution levels in high-income countries, global trends show a limited annual reduction in PM_2.5_ concentration. The analysis highlights disparities between regions, with low- and middle-income countries bearing the brunt of air pollution-related CVDs. In 2019 alone, ambient air pollution was responsible for approximately 4.2 million deaths worldwide. Of these, 70% were caused by CVDs, with approximately 1.9 million deaths from ischemic heart disease and 900,000 deaths from stroke. Policy gaps remain a challenge, with many countries lacking adequate legally binding air quality standards.

We recommend the adoption of WHO air quality guidelines, enhanced monitoring of air pollution levels, and increased investment in interdisciplinary research to understand the full scope of air pollution’s effects on cardiovascular health. Addressing the global cardiovascular crisis linked to air pollution will require coordinated efforts from policymakers, healthcare systems, and global health organisations.

## Introduction

Air pollution exerts a huge toll on human health (1). Recently (2021 data), ambient (outdoor) particulate matter was ranked as the leading risk factor for disability-adjusted life years (DALYs) among all environmental and occupational risks and the third risk factor for cardiovascular diseases (CVDs) associated DALYs after high blood pressure and high low-density lipoprotein (2,3). The global mortality linked to outdoor and indoor (household) air pollution combined has been estimated to be between seven and eight million deaths each year (3), representing more deaths than all wars, malaria, tuberculosis, human immunodeficiency virus (HIV), and other infectious diseases combined (4). Research has highlighted how continuous exposure to air pollution increases the risk of developing disease earlier in our lives (5). The global cost of health damages associated with exposure to air pollution is estimated to be US$8.1 trillion, equivalent to 6.1% of the global gross domestic product (GDP), with 1.2 billion annual workdays lost (6). Predictions indicate that global air pollution-related healthcare costs will surge from US$21 billion in 2015 to US$176 billion in 2060 (7).

Associations have been found between air pollution exposure and adverse effects on almost all organs of the body, including the cardiovascular system (8). CVDs remain the leading cause of global mortality, causing significant morbidity and a large economic burden on the health system (9,10). The detrimental effects of air pollution on cardiovascular health are substantial, worsening major CVDs such as ischemic heart disease (IHD) and stroke (11,12). A number of air pollutants are linked to the incidence, development and exacerbation of CVDs, such as particulate matter (PM), nitrogen dioxide (NO_2_), ozone (O_3_), sulphur dioxide (SO_2_), and black carbon (BC) (13). Particulate matter with a diameter of less than 2.5 micrometres (PM_2.5_) is the pollutant most consistently linked to adverse health effects, especially in regard to the cardiovascular system. Mechanisms encompass endothelial dysfunction, oxidative stress, a reduction in the availability of endothelial-derived nitric oxide, an increase in vasoconstrictive mediators, platelet activation, impaired fibrinolysis, inflammation of endothelial cells, and the activation of inflammatory pathways within these cells (13,14). Long-term exposure to air pollution can accelerate the development of atherosclerosis and promote the instability of plaques within the arteries (15,16,17).

Here we summarise the evidence and analysis underlying the World Heart Report 2024 (18) which provides an overview and re-appraisal of the interplay between air pollution and cardiovascular diseases. The report explores trends in PM_2.5_, the impact of air pollution on cardiovascular health, and policy efforts to reduce these harms, in order to guide policymakers in identifying and addressing the most critical facets of air pollution to improve cardiovascular health worldwide.

## Methods and Data

In order to re-appraise the evidence between air pollution and CVD, here we used the most recently available (2019) data from the World Health Organization (WHO) for PM_2.5_ and attributable burden (3). These data have been extensively described elsewhere (19). Data for the policy analysis were extracted from the United Nation Environment Programme (UNEP) report *Regulating Air Quality: The First Global Assessment of Air Pollution Legislation* (20). Data sources were selected according to key criteria: well-utilised and considered the most valid and reliable; based on robust methodology; provided data according to sex and country, and for attributable burden be available separately for indoor and outdoor air pollution, ultimately allowing more specific recommendations to be made.

In addition, a literature search was conducted in PubMed to identify meta-analyses published between 2010 and 2024 focused on studies examining the relationship between air pollution and CVD outcomes. Keywords included terms related to both air pollution and cardiovascular diseases.^1^ Of the 127 papers identified 42 were included. Data were extracted for both short-term and long-term effects of air pollution on various CVD outcomes, including CVD mortality, heart failure, stroke mortality, myocardial infarction risk, CVD incidence, ischemic heart disease (IHD), and stroke incidence. Pollutants included black carbon (BC), carbon monoxide (CO), nitrogen dioxide (NO_2_), ozone (O_3_), sulphur dioxide (SO_2_), particulate matter (PM_10_ and PM_2.5_), and nitrogen oxides (NO_X_).

## Results

### Levels of ambient air pollution worldwide

#### PM_2.5_ levels

**Figure 1:**
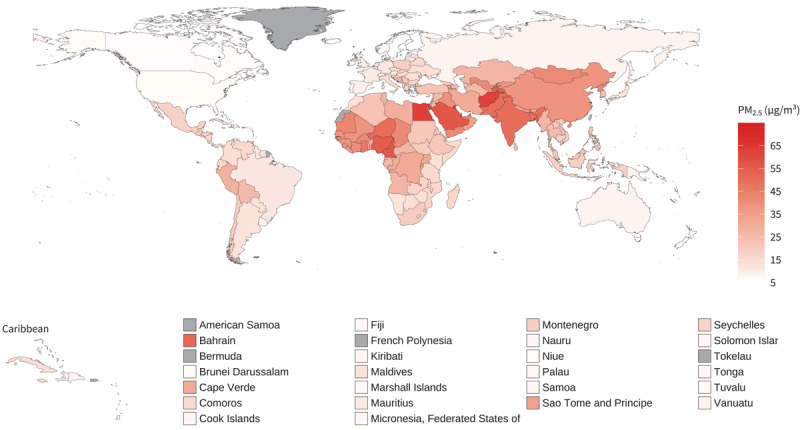
Mean annual PM_2.5_, 2019. Source: https://www.who.int/data/gho/data/indicators/indicator-details/GHO/concentrations-of-fineparticulate-matter-(pm2-5).

In 2019, no country in the world had an average annual PM_2.5_ concentration below the air quality guidance level of 5 µg/m^3^ set by WHO in 2021 (19) (Figure 1). Regional levels in 2019 were grouped in three clusters, with South-East Asia and Eastern Mediterranean regions recording an average annual PM_2.5_ concentration around 43.0 µg/m^3^, Africa and Western Pacific regions around 33.0 µg/m^3^, and Europe and the Americas with values respectively of 12.1 µg/m^3^ and 14.9 µg/m^3^. In the same year, levels of PM_2.5_ were up to three times higher in the South-East Asia and Eastern Mediterranean regions than those observed in the Americas (Table 1). The highest levels of annual average concentrations of PM_2.5_ were recorded in the Global South – Kuwait (64.1 µg/m^3^; 95% Confidence Interval (CI) 55.7–72.5), Afghanistan (62.5 µg/m^3^; CI 45.0–86.5), and Egypt (63.2 µg/m^3^; CI 40.4–92.3). Countries with the lowest levels of concentration were the Bahamas (5.2 µg/m^3^; CI 3.8–7.1), Finland (5.5 µg/m^3^; CI 5.2–5.8), Iceland (5.8 µg/m^3^; CI 5.1–6.5), and Sweden (6.0 µg/m^3^; CI 5.7–6.2).

**Table 1:** PM_2.5_ Levels: lowest and highest 3 countries by WHO Regions, 2019.


WHO REGION	LOWEST		HIGHEST	
	
	MEAN [95% CI]		MEAN [95% CI]	

Africa	Mauritius	10.48 [8.25–12.84]	Niger	50.15 [21.92–99.90]

	Namibia	11.81 [6.32–18.88]	Nigeria	55.64 [37.64–76.88]

	Kenya	12.52 [7.80–17.78]	Cameroon	56.37 [39.06–79.81]

Americas	Bahamas	5.20 [3.77–7.05]	El Salvador	22.15 [14.49–31.82]

	Canada	6.39 [6.21–6.59]	Bolivia (Plurinational State of)	25.23 [16.41–36.37]

	USA	7.18 [7.07–7.28]	Peru	29.07 [22.20–38.42]

Eastern Mediterranean	Morocco	13.44 [11.03–16.52]	Afghanistan	62.49 [45.04–86.46]

	Somalia	14.28 [7.65–24.25]	Egypt	63.16 [40.38–92.33]

	Djibouti	19.98 [7.70–41.73]	Kuwait	64.08 [55.65–72.49]

Europe	Finland	5.47 [5.16–5.76]	Kyrgyzstan	37.58 [26.16–52.31]

	Iceland	5.79 [5.11–6.46]	Uzbekistan	40.98 [29.83–57.12]

	Sweden	5.96 [5.70–6.22]	Tajikistan	53.65 [38.18–76.75]

South-East Asia	Maldives	13.00 [10.33–16.7]	Dem People’s Republic of Korea	41.46 [31.42–53.61]

	Indonesia	19.34 [16.76–23.72]	Bangladesh	45.99 [41.65–51.00]

	Timor-Leste	20.47 [9.02–42.19]	India	50.17 [47.87–52.43]

Western Pacific	Niue	6.74 [3.15–13.16]	Republic of Korea	24.04 [23.39–24.75]

	Tuvalu	6.81 [2.59–13.49]	China	38.15 [36.69–39.42]

	Brunei Darussalam	6.86 [5.76–8.32]	Mongolia	41.30 [30.68–53.50]


**Source:** https://www.who.int/data/gho/data/indicators/indicator-details/GHO/concentrations-of-fine-particulate-matter-(pm2-5).

#### Trends in air pollution over time

**Figure 2:**
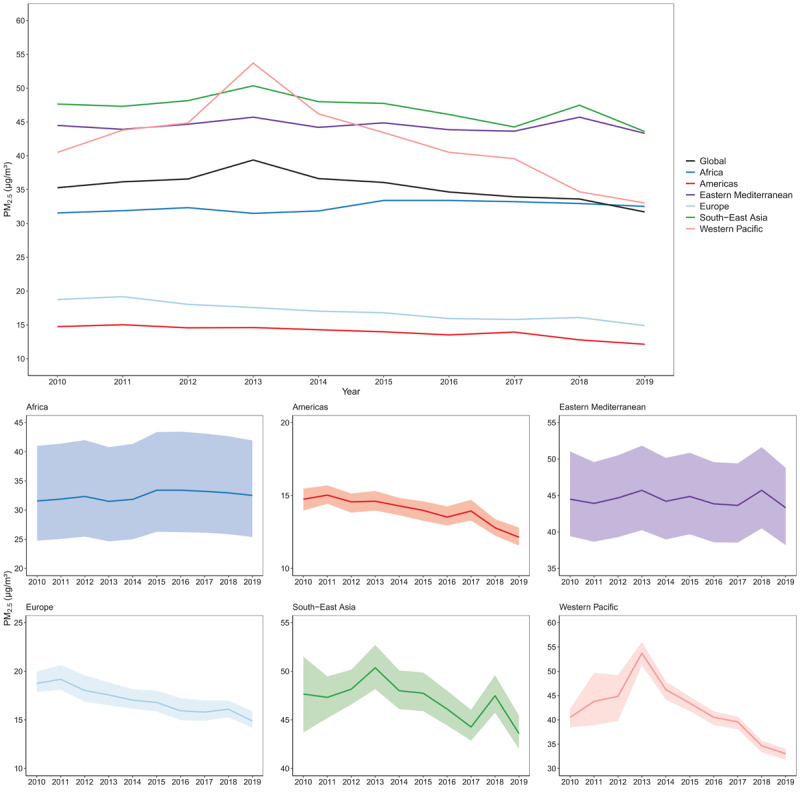
Trends in mean annual PM_2.5_ concentrations by WHO regions, 2010–2019. Note: The shaded areas show uncertainty in estimates. Source: https://www.who.int/data/gho/data/indicators/indicator-details/GHO/concentrations-of-fine-particulate-matter-(pm2-5).

Over the past decade PM_2.5_ levels across regions have remained largely unchanged (Figure 2), with an annual global reduction of just 1.0% between 2010 and 2019 (from 35.3 µg/m^3^ in 2010 to 31.7 µg/m^3^ in 2019). Regionally, the largest decline was recorded in Europe (2.1% average annual change), while PM_2.5_ levels increased annually by 0.3% in Africa. Uncertainty in trends highlights the heterogeneity in data availability with regions of the world being characterised by very sparse and limited data, as in Africa and Eastern Mediterranean regions.

At country level, over the past decade the absolute change in the average annual PM_2.5_ concentration has been heterogeneous across the world with 14% of the countries experiencing large declines (between 5–10 ug/m^3^), and others showing no change or increases. All countries in the Europe and South-East Asia regions have experienced a decline in the level of air pollution between 2010 and 2019, while during the same period an increase in the average annual PM_2.5_ concentration was observed for 60% of the countries in Africa. In the Eastern Mediterranean, Americas, and Western Pacific regions, 36%, 46%, and 45% of countries respectively, recorded an increase in the level of PM_2.5_ concentration. Countries with the largest absolute decrease were Tajikistan (9.8 ug/m^3^), the North Macedonia (9.4 ug/m^3^), and China (9.0 ug/m^3^), while the larger absolute increase was observed in Mauritania (3.1 ug/m^3^) and Sierra Leone (3.1 ug/m^3^). The largest relative declines have been observed in most European countries (between 25% and 30% relative change), while the largest relative increases are recorded in Angola (12.2%), Cabo Verde (10.3%), Liberia (8.9%), Sierra Leone (8.5%), and Palau (8.2%).

### Burden of cardiovascular mortality and morbidity attributable to ambient air pollution: levels and trends

#### Overall mortality attributed to PM_2.5_

**Figure 3:**
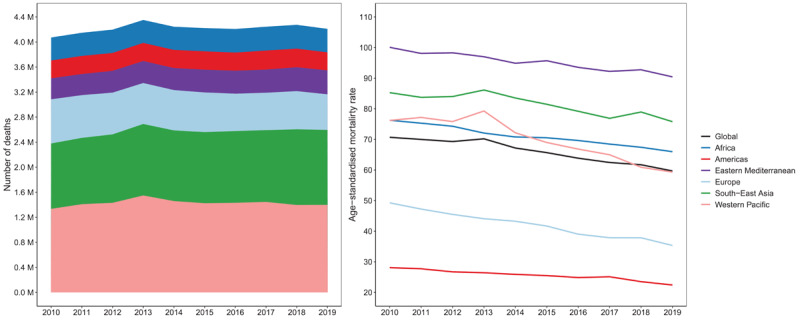
Number of all causes deaths attributable to ambient air pollution (left panel) and age-standardised mortality rates (per 100,000 people) attributable to ambient air pollution (right panel) for both sexes and by regions.

In line with the overall fall in PM_2.5_ globally, between 2010 and 2019, the all-cause age-standardised death rate, attributable to air pollution, declined globally from 70.7 deaths per 100,000 people to 59.7 deaths per 100,000 people, with all regions experiencing an average annual decline between 1% and 3% (Figure 3). However, the actual numbers of deaths increased over the same period. In 2019, ambient air pollution caused 4.2 million deaths and over 100.4 million DALYs globally. The number of deaths in 2019 was almost 140,000 more than the number recorded in 2010, a rise mostly driven by the South-East Asia (152,000 more deaths), Western Pacific (64,000 more deaths), and Eastern Mediterranean (47,000 more deaths) regions. Europe is the only region which recorded over 135,000 fewer deaths in 2019 compared to 2010.

#### Cardiovascular burden attributed to PM_2.5_

Of the 4.2 million deaths globally attributed to ambient air pollution in 2019, 70% were caused by cardiovascular conditions, notably ischaemic heart disease (1.9 million deaths) and stroke (900,000 deaths).

In 2019, the number of air pollution attributable deaths from IHD and stroke was higher in the Western Pacific (957,000 deaths) and South-East Asia (762,000 deaths) regions, to which China and India – the two largest countries in the world – belong. Overall, nearly 30% of all global ambient air pollution IHD deaths were recorded in the Western Pacific region, while the lowest percentages were observed in Africa and the Americas.

The global number of IHD deaths attributable to air pollution increased almost 200,000 from 2010 to 2019. The levels of age standardised IHD mortality rates (deaths per 100,000 people) attributable to air pollution remained mostly stable or showed a very limited decline from 2010–2019 across regions, except for Europe that experienced a decline of 28% over the entire period. Most regions experienced an increase in actual numbers of IHD deaths between 20%–27% in this period, except for the Americas and Europe, where the number of IHD deaths attributable to air pollution increased 2.4% and decreased 19.2% respectively.

**Figure 4:**
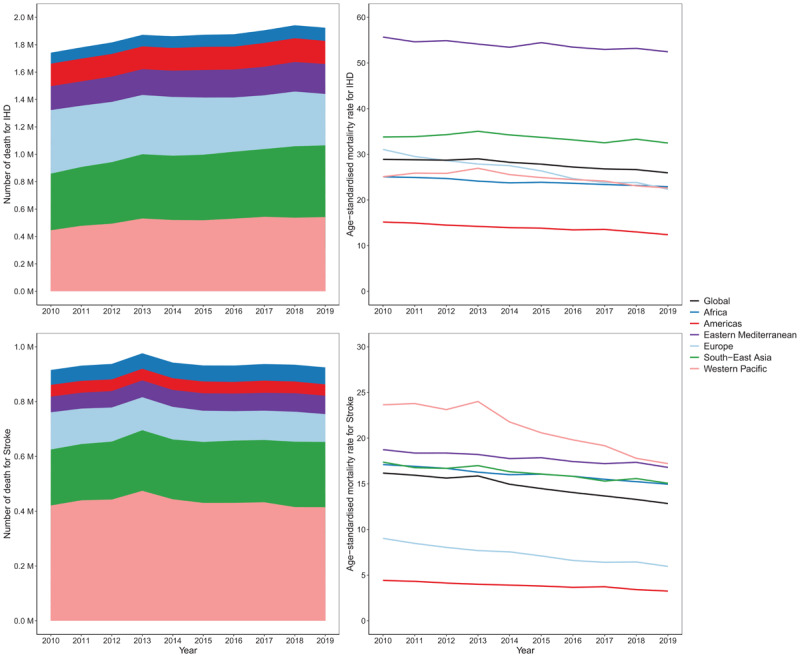
Number of IHD and stroke deaths attributable to ambient air pollution (left panels) and age-standardised mortality rates (per 100,000 people) attributable to ambient air pollution (right panels) for both sexes and WHO regions.

Levels of age-standardised stroke mortality rates attributable to air pollution declined in all regions from 2010–2019, with the Eastern Mediterranean, Africa, and South-East Asia regions recording an average annual reduction around 1%. The remaining regions experienced annual average reductions of 2.6–3.3% (Figure 4). The actual numbers of deaths from stroke attributable to air pollution globally increased only 1% from 2010–2019, though with significant regional variation. Increases were observed in Africa, South-East Asia, and Eastern Mediterranean regions in the period, while Europe experienced a 25.3% decline, and the Americas and Western Pacific regions saw lesser reductions (Figure 4).

**Figure 5:**
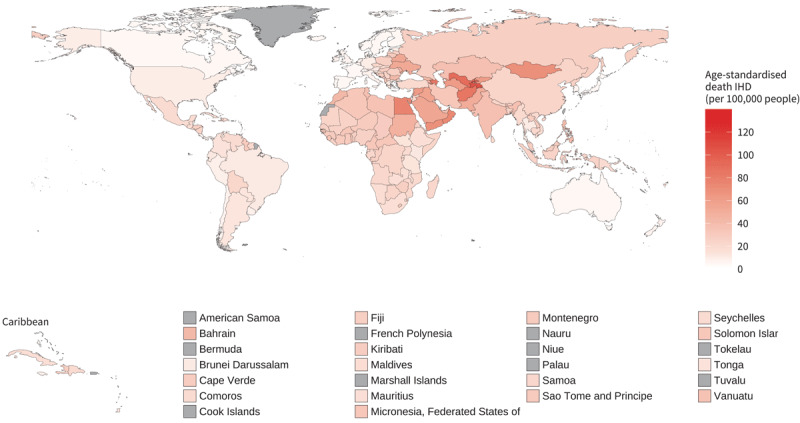
Age-standardised ischemic heart disease mortality rates (per 100,000 people) attributable to ambient air pollution for both sexes, 2019. Source: https://www.who.int/data/gho/data/indicators/indicator-details/GHO/ambient-air-pollution-attributable-death-rate-(per-100-000-population-age-standardized).

**Figure 6:**
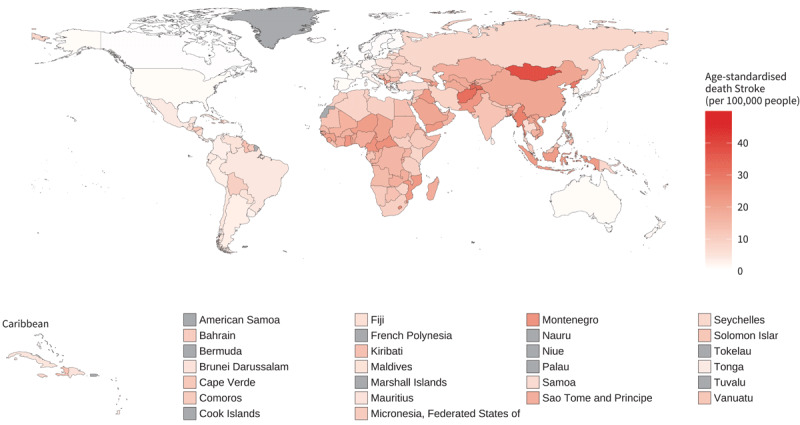
Age-standardised stroke mortality rates (per 100,000 people) attributable to ambient air pollution for both sexes, 2019. Source: https://www.who.int/data/gho/data/indicators/indicator-details/GHO/ambient-air-pollution-attributable-death-rate-(per-100-000-population-age-standardized); https://www.who.int/data/gho/data/indicators/indicator-details/GHO/household-air-pollution-attributable-death-rate-(per-100-000-population-age-standardized).

The World Health Organization has highlighted the disproportionate impact of air pollution on low- and middle-income countries (LMICs), where the reduction in age-standardised mortality rates due to CVDs is slower compared to high-income countries (HICs). This is confirmed by 2019 data showing that countries in the global south are the most affected by ambient air pollution in terms of attributable mortality due to ischaemic heart diseases (IHD) and stroke (Figure 5 and Figure 6). In 2019, the three countries with the highest age-standardised IHD mortality per 100,000 people attributable to ambient air pollution were Tajikistan (106 per 100,000 people; CI 74–134), Uzbekistan (90 per 100,000 people; CI 61–116), and Afghanistan (84 per 100,000 people; CI 60–106). The lowest levels were recorded in Norway (4.8 per 100,000 people; CI 2.4–7.0), Portugal (5.2 per 100,000 people; CI 2.8–7.4), and France (5.4 per 100,000 people; CI 3.1–7.3). In the same year the three countries with the highest stroke ambient air pollution attributable mortality were Mongolia (38 per 100,000 people; CI 21–58), Tajikistan (33 per 100,000 people; CI 18–52), and Afghanistan (32 per 100,000 people; CI 17–50). While Canada (0.86 per 100,000 people; CI 0.24–1.84), Iceland (0.87 per 100,000 people; CI 0.21–2.00), and Norway (0.99 per 100,000 people; CI 0.28–2.10) in the Americas and Europe regions recorded the lowest attributable death.

In 2019, IHD DALYs attributable to ambient air pollution resulted in 1973 DALYs per 100,000 people in Afghanistan (CI 1404–2500) and Tajikistan (CI 1339–2433), and almost 1700 per 100,000 people in Uzbekistan (CI 1154–2185). The lowest levels of IHD DALYs attributable to ambient air pollution were recorded in Norway (81 per 100,000 people; CI 41–120), Sweden (91 per 100,000 people; CI 49–144), and Finland (97 per 100,000 people; CI 47–150), where the lowest concentration levels of PM_2.5_ exist. For stroke, ambient air pollution was responsible for 927 DALYs per 100,000 people in Mongolia (CI 510–1410), 738 per 100,000 people in Afghanistan (CI 399–1172), and 689 per 100,000 people in the Democratic People’s Republic of Korea (CI 370–1060); whereas Iceland (17 per 100,000 people; CI 4–39), Sweden (21 per 100,000 people; CI 6–47), and Canada (21 per 100,000 people; CI 6–45) recorded the lowest levels of ambient air pollution attributable DALYs.

#### Associations between air pollutants and different cardiovascular diseases

**Figure 7:**
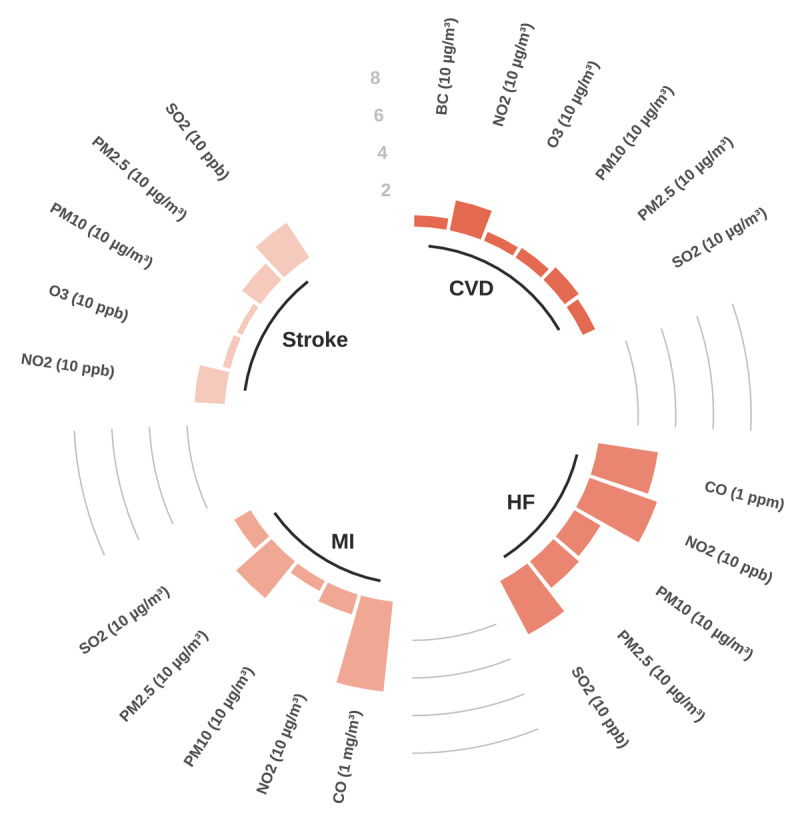
Short-term exposure: Percentage increase in the risk of cardiovascular diseases (selected outcomes) by type of air pollutant. Note: Each bar represents the percentage increase in the risk of developing the condition due to short-term exposure to specific pollutants (increase unit in pollutant provided in brackets in figure). **CVD** – Cardiovascular disease mortality; **HF** – Overall risk of heart failure incidence, mortality, and hospitalization; **MI** – Myocardial infarction incidence; **Stroke** – Stroke mortality; **BC** – Black carbon. Source: See Appendix Table 1 and Table 2 for the full set of data.

**Figure 8:**
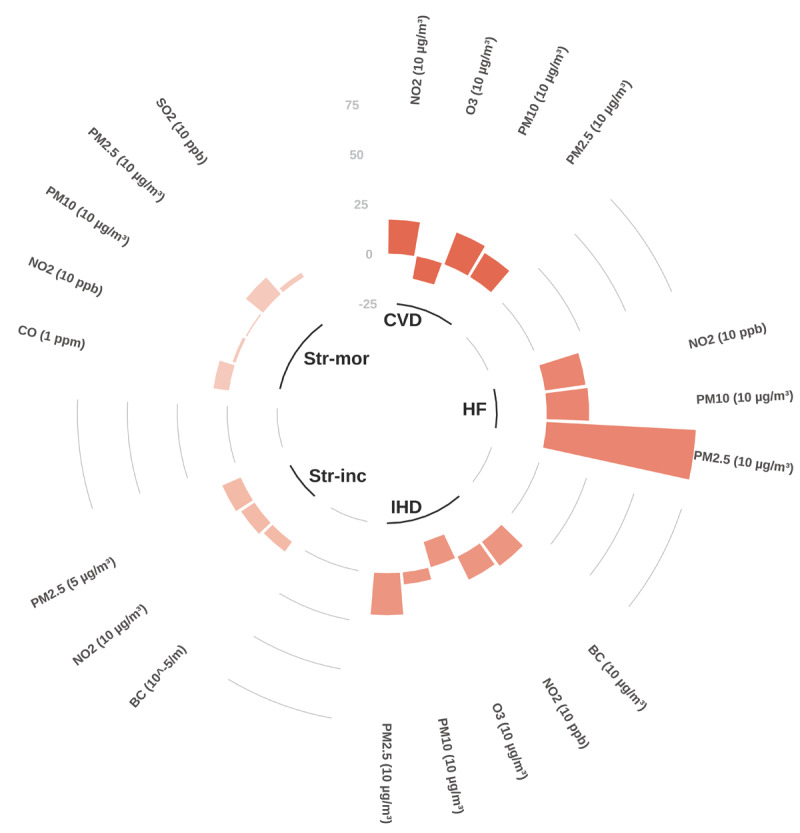
Long-term exposure: Percentage increase in the risk of cardiovascular diseases (selected outcomes) by type of air pollutant. Note: Each bar represents the percentage increase in the risk of developing the condition due to long-term exposure to specific pollutants (increase unit in pollutant provided in brackets in figure). **CVD** – Cardiovascular disease mortality; **HF** – Overall risk of heart failure incidence, mortality, and hospitalization; **IHD** – Ischaemic heart disease mortality; **Str-inc** – Stroke incidence; **Str-mor** – Stroke mortality; **BC** – Black carbon. Source: See Appendix for the full set of data.

There is expansive literature linking air pollution to most cardiovascular conditions, albeit most studies use data from high-income countries and limited data from low- and middle-income countries. Associations have been found between various air pollutants and coronary artery disease, cerebrovascular disease, stroke, heart failure, cardiac arrhythmia and arrest, venous thromboembolism, and peripheral artery disease, as well as more limited evidence on CVD conditions such as pulmonary hypertension, dilated cardiomyopathy, congenital heart disease, dyslipidaemia, and others (21). Review of large-scale meta-analyses found robust positive associations between both short- (Figure 7) and long-term (Figure 8) exposure to criteria air pollutants and most major cardiovascular conditions, including IHD, stroke, heart failure and myocardial infarction. In many cases, various CVDs are associated with more than one pollutant. The risk estimates for even single pollutants are substantial, especially for long-term exposure (e.g. risk ratios mostly greater than 10% increase).

#### Indoor/household air pollution

**Figure 9:**
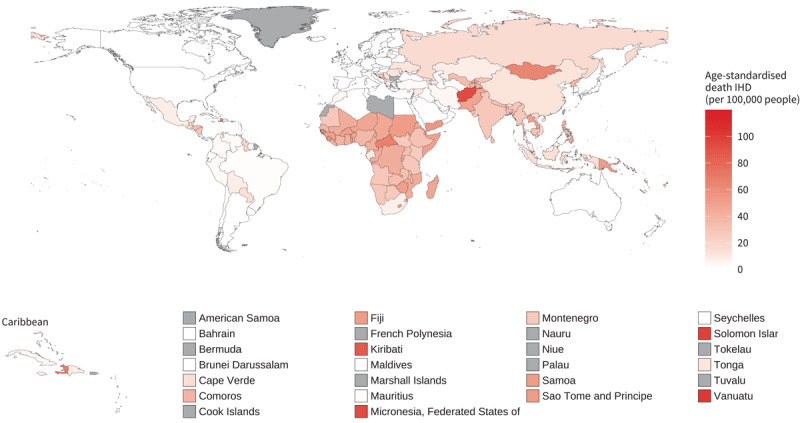
Age-standardised ischaemic heart disease mortality rates (deaths per 100,000 people) attributable to household air pollution for both sexes, 2019.

**Figure 10:**
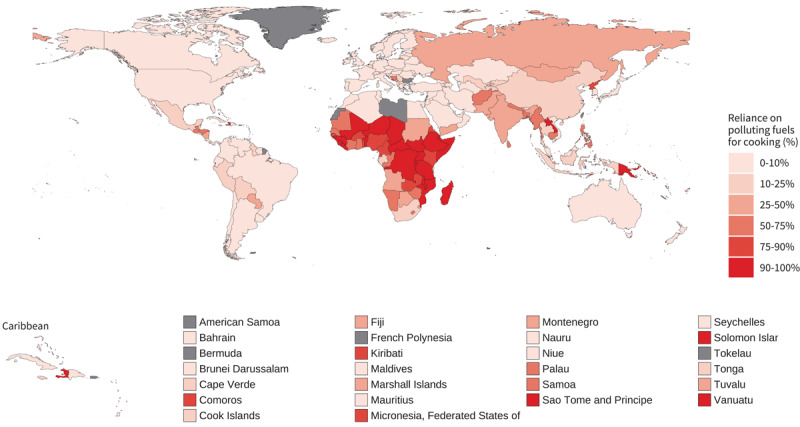
Proportion of population with primary reliance on polluting fuels and technologies for cooking (%), 2021. Source: https://www.who.int/data/gho/data/indicators/indicator-details/GHO/gho-phe-population-with-primary-reliance-on-polluting-fuels-and-technologies-for-cooking-proportion.

The burden of CVD mortality resulting from exposure to household (indoor) air pollution from polluting fuel used for cooking and heating (solid fuels such as wood, coal, animal dung, charcoal, and crop wastes, and also kerosene) remains a major challenge especially in low and middle-income countries. In 2019, the three countries with the highest age-standardised IHD mortality attributable to household air pollution (deaths per 100,000 people) were Vanuatu (103; CI 79–126), Solomon Islands (100; CI 77–122), and Federal State of Micronesia (94; CI 69–118). The lowest levels, outside of the high-income countries where no burden for household air pollution is estimated (due to limited solid fuel use), were recorded in Argentina (0.3; CI 0.0–2.6), Jordan (0.4; CI 0.0–3.2), and Tunisia (0.8; CI 0.0–5.0) (Figure 9). In 2021 the proportion of population with primary reliance on polluting fuels and technologies for cooking, was highest in Africa (Sub-Saharan region) with South Sudan (100%; CI 96.1–100), Burundi (99.8%; CI 94.6–100), and Liberia (99.6%; CI 94.6–100) recording the highest values (Figure 10).

#### Global inequalities in outdoor and household air pollution

**Figure 11:**
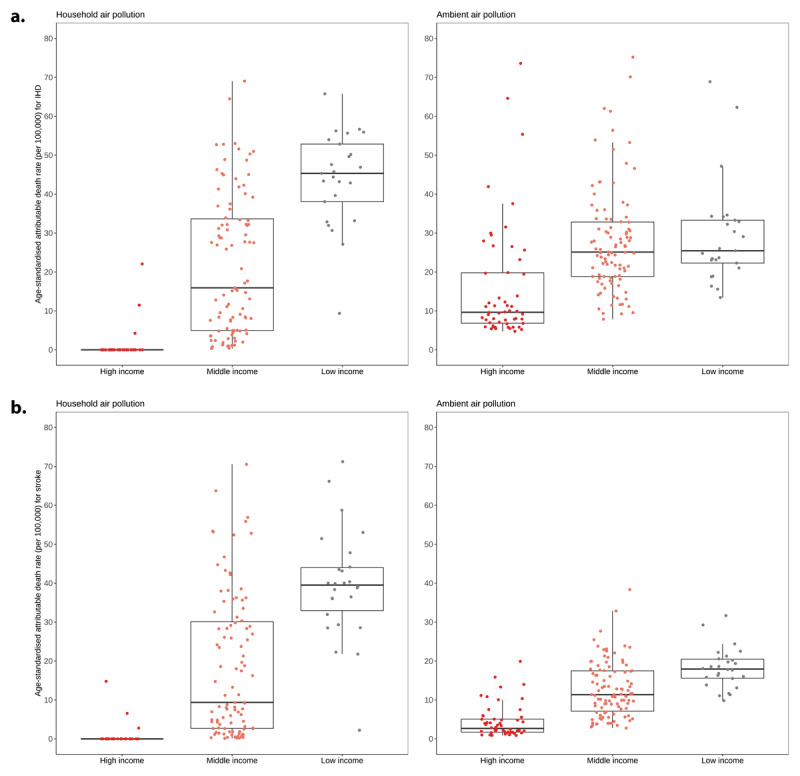
Ischaemic heart disease and stroke age-standardised mortality rates (deaths per 100,000 people) attributable to household air pollution (a) and ambient air pollution (b), by income level for both sexes, 2019. Source: https://www.who.int/data/gho/data/indicators/indicator-details/GHO/household-air-pollution-attributable-death-rate-(per-100-000-population-age-standardized).

When countries are grouped by income level, the impact of ambient (outdoor) and household air pollution are markedly different (Figure 11). Countries in the low-income group experience higher levels of age-standardised stroke and IHD attributable mortality due to both ambient and household air pollution than those in the middle- and high-income groups, with the exception of IHD mortality attributable to ambient air pollution where the trend was overlapping. More marked differences were observed in levels of stroke and IHD mortality attributable to household air pollution compared to ambient air pollution.

#### Policies and air quality guidelines

**Figure 12:**
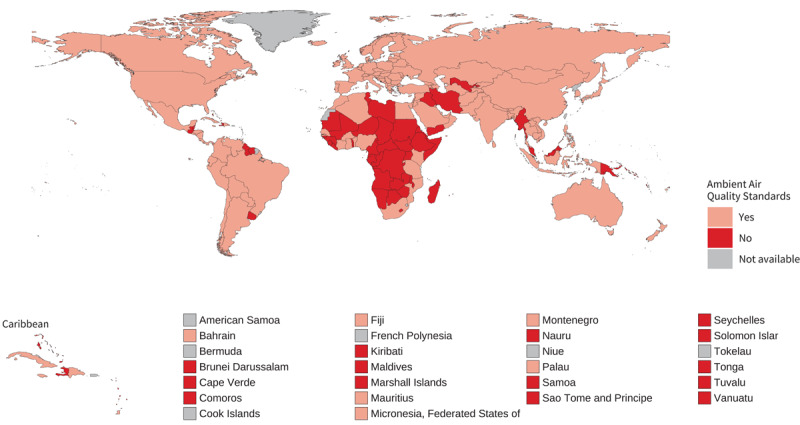
Countries with legal instruments containing Ambient Air Quality Standards. Source: https://www.unep.org/resources/report/regulating-air-quality-first-global-assessment-air- pollution-legislation.

In 2021, the WHO updated its Global Air Quality Guidelines, significantly lowering the recommended threshold for annual average PM_2.5_ concentrations to 5 µg/m^3^ from the previous level of 10 µg/m^3^ (22). In the same year, UNEP assessed national air quality legislation and found that only 122 countries (64%) had ambient air quality standards (AAQS) embedded in their legislation or policy guidelines (20). The majority of countries without AAQS legislation are in the African region (Figure 12). Moreover, UNEP highlighted that 55% of countries permit regular exceedances of their legal standards, which can obscure instances of non-compliance, particularly when these exceedances are broadly defined.

Emerging guidelines (e.g. WHO) are beginning to address pollutants such as black carbon, dust from sandstorms, and ultrafine particles, given their growing recognition as significant contributors to adverse health effects. However, these pollutants are only sporadically included in national air quality standards, and few countries actively monitor or regulate them.

## Discussion

This article presents a summary of World Heart Report 2024 on the relationship between air pollution and cardiovascular diseases, highlighting significant disparities across regions. Our findings revealed the significant burden of CVD attributable to PM_2.5_; concentrations of which remain high despite gradual improvements in air quality in some regions. Some regions show increases in PM_2.5_ and an accompanying cardiovascular burden, especially in many LMICs where the impact will be confounded by other risk factors for CVD and lower resilience in terms of tackling air pollution and access to high quality healthcare. Furthermore, assessment of policies reveals clear deficiencies in air quality strategies in many regions, especially in terms of legally binding targets for air pollutants. Given the prevalence of CVDs globally, this assessment highlights some of the global challenges posed by air pollution and stresses the urgency of addressing this global health crisis.

Variations in the level of air pollution across regions significantly impact the burden of cardiovascular disease. The Western Pacific and South-East Asia regions, particularly China and India, experience the highest number of air pollution-related IHD and stroke deaths. However, countries in the global south, especially low and low-middle income countries bear the heaviest burden of air pollution related CVD. Also, high-income countries, while experiencing lower levels of PM_2.5_, should not become complacent, as health effects are evident even below the most stringent air quality guidelines (23).

Age-standardised mortality from PM_2.5_ is falling (somewhat) in line with the slow reductions in PM_2.5_ levels globally. However, due to a growing and ageing global population, the actual numbers of CVD deaths attributed to air pollution are increasing. The headline figures are that the air pollutant PM_2.5_ is linked to 1.9 million deaths from IHD and 900,000 deaths from stroke. However, our analysis focused on a single air pollutant and just two major cardiovascular conditions. Review of meta-analyses encompassing additional air pollutants and found robust associations with many criteria air pollutants and all major CVDs. While we did not look at the effects of multiple pollutants in our models, there will be some overlap in risk estimates from closely related pollutants. Nonetheless, there are certainly compounded risks for the combined air pollutant mixture, and clear interactions with other cardiovascular and environmental risk factors (see full World Heart Report 2024 for further discussion (18)). Looking beyond meta-analyses, individual studies can be found presenting evidence for associations between various air pollutants and an extremely broad range of cardiovascular conditions. Thus, the estimates of cardiovascular burden outlined in this paper, while already staggering, are likely to be considerable underestimates of the true burden.

The bulk of air pollution research focuses on outdoor air pollution, however, there are many sources of indoor air pollution. Levels of air pollution can build up in poorly ventilated indoor environments and exposure may be very high given the high proportion of time spent indoors. The World Health Organization estimates that over 2 billion people worldwide rely on polluting fuels such as wood, coal, crop waste, animal dung, or charcoal paired with inefficient stoves for cooking (24). Considerable evidence increasingly indicates the role of household air pollution in contributing to a broad range of cardiorespiratory, paediatric, and maternal conditions, with these disease categories specifically highlighted by WHO (25). In 2019, household air pollution contributed to 3.2 million deaths annually, of which half of these were due to cardiovascular diseases, with 1 million from IHD and 700,000 from stroke. Exposure to household air pollution is now among the top ten risk factors for disease, with the poorest communities in low- and middle-income countries (LMICs) most affected. Furthermore, there may be age, gender and occupational differences in both levels and type of exposure, and these are not adequately captured with current epidemiological methods to attribute exposure to individuals based on residential address or domestic fuel use. Studies are needed that measure indoor and occupational exposure to air pollutants, especially in LMIC settings where individual differences in exposure may be greatest, to ascertain the health consequences of these demographic factors, their impact on inequalities and how air quality interventions may tackle them. There is also a need to explore the health effects of indoor sources of air pollution that arise from other sources than solid fuel combustion, including in high income settings.

Achieving universal access to clean fuels and technologies is a key element of the UN Sustainable Development Goals (26). Government intervention via policy and targeted investments can accelerate the adoption of clean cooking solutions. This has been shown to be successful in Asian countries including China, India, and Indonesia (27,28,29). A direct transition to clean fuels remains the ideal solution. However, in many regions, particularly across LMIC settings, progress remains slow. Acknowledging these challenges, WHO guidelines recommend expansion of the use of clean fuels and technologies including solar, electricity, biogas, liquefied petroleum gas (LPG), natural gas, and alcohol fuels. It should be noted that while the longer-term goal should be a transition away from non-renewable fuels, in the short-term the use of LPG should be promoted as a scalable, transitional clean household energy solution. Where access to clean fuels remains limited, more advanced combustion cookstoves that meet the emission targets in the WHO Guidelines may be prioritised in the transition to clean cooking solutions.

Policy initiatives at various levels have shown promise in improving air quality. However, there is considerable variation in the implementation and effectiveness of these policies across different regions. For instance, while some cities have successfully implemented measures to reduce air pollution from transportation and industry, national legislation often lags behind, with only 64% of countries having ambient air quality standards that meet WHO guidelines. Emerging policies, such as the WHO’s ‘best practice’ recommendations for black carbon, ultrafine particles (UFPs), and desert dust, offer a forward-looking approach to tackling air pollution (22), although it should be borne in mind that these pollutants cannot be systematically monitored at scale at present.

While beyond the scope of the current paper, other emerging policy considerations from a variety of sectors that will impact on PM levels should be considered. These include agriculture; a significant source of ammonia which is a precursor for secondary particulate matter, as well as the generation of combustion-derived air pollution (and green-house gases) from crop and biomass burning. The transport sector is a significant source of PM_2.5_, NO_2_, and ultrafine particles, the latter of which may have a disproportionate effect on the cardiovascular system due to their complex composition and ability to penetrate into the blood (13). Significant progress has also been made in reducing tailpipe emissions from new vehicles, particularly in terms of NO_2_ and particulate matter, with many countries enforcing regular vehicle checks to ensure compliance. The emissions, and health effects, of non-exhaust particulate matter (e.g. from brake and tyre wear) will be an important legislative consideration in future years. While progress has been made in reducing transport pollution and exposure to industrial sources of pollution in some regions, it is important not to overlook these seemingly ‘older air quality issues’, especially in LMICs where average vehicle age is greater and polluting industries can still reside next to population-dense cities. Finally, the relationship between air pollution and climate change adds another layer of complexity. Both issues share common sources, such as fossil fuel combustion, and addressing one often benefits the other. Nonetheless, it will be important to ensure that interventions for either climate change or air pollution are rigorously tested by scientific methods to minimise inadvertent consequences on the other.

In discussing global efforts to reduce air pollution and its impact on cardiovascular health, several case studies highlight successful interventions across diverse regions. Some illustrative examples include Barcelona, Spain, which achieved a 30% reduction in NO_2_ emissions by implementing policies targeting ‘green’ urban zones, that provide safe active travel routes in urban spaces and reduce vehicle congestion. Measures included the employment of new air quality monitoring stations, the creation of a new bus network, the expansion of the metro and bicycle lane networks (resulting in 56% more bicycle journeys) and the incentivization of journeys on foot, with more pedestrian friendly streets and spaces (30). Similarly, Ulaanbaatar, Mongolia, significantly decreased winter PM_2.5_ levels by phasing out raw coal for cleaner energy sources (31). In Cameroon, grassroots advocacy and government collaboration have raised awareness and driven educational campaigns to combat air pollution’s health effects. Buenos Aires, Argentina, has employed comprehensive urban planning to reduce air pollution and promote sustainability, aiming for reduction in greenhouse gases by 84% by the year 2050 (32). Polycentric urban design, where residents can meet all their daily needs (work, home, amenities, food, health, education, culture recreation, and sports) within short periods of walking or other active travel, would also reduce traffic related emissions. Lastly, Beijing, China, has demonstrated the effectiveness of large-scale governmental action, having achieved a 35% reduction in PM_2.5_ concentrations through stringent emissions standards and industrial restructuring (33).

## Recommendations

All countries and stakeholders must urgently work together to accelerate efforts to curb air pollution levels and implement policy and health interventions to protect people from its most harmful effects. These actions will be critical to achieving Sustainable Development Goals related to cutting non-communicable disease mortality, as well as having broader benefits with regard to tackling the climate crisis.

To help promote urgent action against air pollution and its impacts on cardiovascular disease and health more broadly, the World Heart Federation (WHF) recommends the following:

### Primary recommendations

1. All countries must adopt the new WHO global guidelines on air quality. This includes making a roadmap of strategies to meet the interim targets outlined by WHO whilst progressing to the overall guideline level. Policies should be multifaceted and multi-sectoral, encompassing, among others, health, housing, city design, transport, and agriculture.
2. WHF supports the implementation of a global fossil fuel non-proliferation treaty. Country commitments to these treaties must be maintained, ideally through legally binding agreements, and suitable implementation strategies must be employed to rapidly reduce the use of fossil fuels.
3. Countries and technical bodies, particularly in LMICs, should urgently improve air pollution monitoring and modelling where there are gaps. This includes expansion of the stationary monitoring network in both rural and urban areas that will help provide more accurate estimates of air pollution levels and trends.
4. Countries, multilaterals, and philanthropies must increase funding into multidisciplinary air pollution research and technological innovations to improve air quality and strategies to implement interventions for reducing air pollution.
5. Health and research agencies at country, regional, and global levels should conduct additional studies into the cardiovascular effects of air pollution and CVDs linked to ambient and household air pollution, in addition to the role of the cardiovascular system in the disease of other organs which are exacerbated by air pollution. This should include the study of the cardiovascular effects of less-well-researched air pollutants, so that policies and interventions can target the air pollutants that are most harmful. This will support the design and implementation of health interventions.

### Secondary recommendations

1. WHF encourages cardiologists, cardiovascular scientists, health practitioners in general, cardiovascular communities and health foundations to advocate for the need to recognize air pollution as a major risk factor for cardiovascular health, engage with stakeholders, and help prioritise resources and political will to tackle this issue.
2. WHF encourages the healthcare sector to take a leading stance in reducing emissions of air pollution as part of sustainability strategies. Currently the healthcare sector accounts for almost 5% of global greenhouse gas emissions.
3. Greater efforts must be made to improve education on the health impacts of air pollution, including at secondary school, undergraduate, and postgraduate levels, for health professionals and through training programmes for disciplines essential for research in the field, e.g., toxicology and epidemiology.
4. More studies on the cardiovascular effects of air pollution are required in LMICs. Where stationary monitoring is limited in LMICs, the use of low-cost monitors and in-situ measurement of health parameters by portable devices would be beneficial to allow more research into the cardiovascular effects of air pollution in these settings.

## Conclusions

In summary, while progress is being made, the burden of air pollution on cardiovascular health remains high. The concerning figures highlighted here reflect the prevalence of cardiovascular disease, the ubiquitous exposure to air pollution and the many ways in which air pollution can impair cardiovascular function and promote CVD. The science behind these associations is robust, with clear mechanistic underpinnings to demonstrate causality. The estimates of cardiovascular burden from poor air quality are shocking, yet are clear underestimates of the true burden. Air pollution represents a public health emergency that can no longer be ignored.

There is an urgent need for global commitment to implement effective interventions and policies to reduce air pollution and realise the potential health benefits. These include enhancing education on the health impacts of air pollution, improving air quality monitoring in low-resource settings, increasing funding for interdisciplinary research, adopting WHO air quality guidelines, and supporting international cooperation for fossil fuel non-proliferation treaties. The health sector must also advocate for decarbonization and sustainable practices within healthcare and beyond. By addressing air pollution and its cardiovascular effects comprehensively, we can make significant strides towards improving global heart health and achieving long-term environmental sustainability.

## Additional File

The additional file for this article can be found as follows:

10.5334/gh.1364.s1 Appendix. Appendix Tables 1 and Table 2.

## Footnotes

(‘Air Pollution/adverse effects’ [MAJR] OR ‘Air Pollution/analysis’ [MAJR] OR ‘Air Pollutants/adverse effects’ [MAJR] OR ‘Air Pollutants/analysis’ [MAJR]) AND (‘Cause of Death’ [Mesh] OR ‘Cardiovascular Diseases’ [Mesh]).
