# Supplementary material for: Clearing the Air to Address Pollution’s Cardiovascular Health Crisis
Source: Glob Heart. 2024 Oct 30;19(1):82. doi: 10.5334/gh.1364 (PMC11523843; doi:10.5334/gh.1364)
Supplement: Appendix. — Appendix Tables 1 and Table 2. [file gh-19-1-1364-s1.pdf]

Appendix

Table of contents

Systematic review - Association between exposure to air pollutants and increased risk of CVDs 1
Appendix Table 1: Full set of meta-analysis data, short-term effect of air pollution 2
Appendix Table 2: Full set of meta-analysis data, long-term effect of air pollution 5

Systematic review - Association between exposure to air pollutants and increased risk of CVDs

A systematic search was conducted in PubMed, to identify meta-analyses published between 2010 and 2024 that investigated the association between air pollution and cardiovascular outcomes. A total of 127 papers were initially identified through the search keywords including: ("Air Pollution/adverse effects"[MAJR] OR "Air Pollution/analysis"[MAJR] OR "Air Pollutants/adverse effects"[MAJR] OR "Air Pollutants/analysis"[MAJR]) AND ("Cause of Death"[Mesh] OR "Cardiovascular Diseases"[Mesh]). Following a screening process, 42 papers remained relevant for further analysis.

Data were extracted for the following outcomes: CVD mortality, heart failure, stroke mortality, and myocardial infarction risk for short-term effects, and CVD incidence, heart failure, ischemic heart disease (IHD), and stroke incidence and mortality for long-term effects. Data were grouped based on short- and long-term effects, as reported in the papers.

Effects of air pollutants, including black carbon (BC), carbon monoxide (CO), nitrogen dioxide (NO_2), ozone (O_3), sulphur dioxide (SO_2), particulate matter with diameters of 10 micrometres or less (PM_{10}), particulate matter with diameters of 2.5 micrometres or less ($PM_{2.5}$), and nitrogen oxides (NOX), were extracted

Significant estimates were identified based on the confidence intervals. In the main paper we report a subset of the data extracted based on the following criteria: 1) we selected significant estimates for each outcome and air pollutant; 2) following step 1 we selected studies with larger samples; 3) following step 2 we selected papers with the largest geographical representation.

All data extracted are reported in Appendix Table 1 and Appendix Table 2.

Appendix Table 1: Full set of meta-analysis data, short-term effect of air pollution

Cardiovascular condition	Pollutant	Study size	Relative Risk	Relative Risk 95% CI	Standard error in 10-	Odds Ratio	Odds Ratio 95% CI	Percentage increase	Percentage increase 95% CI	Increase in pollutant	Study	Coverage
Atrial Fibrillation	CO	7	1.02	(0.987-1.054)						1000 ppb	Int Heart J. 2021 Mar 30;62(2):290-297. doi: 10.1536/ihj.20-523. Epub 2021 Mar 6.	Global
Atrial Fibrillation	NO2	8	1.032	(1.006-1.058)						10 ppb	Int Heart J. 2021 Mar 30;62(2):290-297. doi: 10.1536/ihj.20-523. Epub 2021 Mar 6.	Global
Atrial Fibrillation	O3	7	1.005	(0.996-1.047)						10 ppb	Int Heart J. 2021 Mar 30;62(2):290-297. doi: 10.1536/ihj.20-523. Epub 2021 Mar 6.	Global
Atrial Fibrillation	PM10	7	1.011	(0.998-1.024)						10 µg/m3	Int Heart J. 2021 Mar 30;62(2):290-297. doi: 10.1536/ihj.20-523. Epub 2021 Mar 6.	Global
Atrial Fibrillation	PM2.5	9	1.018	(1.000-1.037)						10 µg/m3	Int Heart J. 2021 Mar 30;62(2):290-297. doi: 10.1536/ihj.20-523. Epub 2021 Mar 6.	Global
Atrial Fibrillation	PM2.5	10				1.11	(1.03-1.19)			10 µg/m3	Sci Total Environ. 2021 Aug 25;784:147106. doi: 10.1016/j.scitotenv.2021.147106. Epub 2021 Apr 15.	Global
Atrial Fibrillation	SO2	8	1.029	(1.003-1.057)						10 ppb	Int Heart J. 2021 Mar 30;62(2):290-297. doi: 10.1536/ihj.20-523. Epub 2021 Mar 6.	Global
Atrial fibrillation prevalence	CO	4				1.02	(0.99-1.06)			1 ppm	Ecotoxicol Environ Saf. 2021 Jan 15;208:111508. doi: 10.1016/j.ecoenv.2020.111508. Epub 2020 Nov 1.	Global
Atrial fibrillation prevalence	NO2	5				1.03	(1.01-1.04)			10ppb	Ecotoxicol Environ Saf. 2021 Jan 15;208:111508. doi: 10.1016/j.ecoenv.2020.111508. Epub 2020 Nov 1.	Global
Atrial fibrillation prevalence	O3	4				1.01	(0.97-1.06)			10ppb	Ecotoxicol Environ Saf. 2021 Jan 15;208:111508. doi: 10.1016/j.ecoenv.2020.111508. Epub 2020 Nov 1.	Global
Atrial fibrillation prevalence	PM10	7				1.03	(1.01-1.05)			10 µg/m3	Ecotoxicol Environ Saf. 2021 Jan 15;208:111508. doi: 10.1016/j.ecoenv.2020.111508. Epub 2020 Nov 1.	Global
Atrial fibrillation prevalence	PM2.5	9				1.01	(1.00-1.02)			10 µg/m3	Ecotoxicol Environ Saf. 2021 Jan 15;208:111508. doi: 10.1016/j.ecoenv.2020.111508. Epub 2020 Nov 1.	Global
Atrial fibrillation prevalence	SO2	4				1.05	(1.01-1.09)			10ppb	Ecotoxicol Environ Saf. 2021 Jan 15;208:111508. doi: 10.1016/j.ecoenv.2020.111508. Epub 2020 Nov 1.	Global
Cardiac Arrhythmia mortality	CO	3	1.077	(0.790-1.467)						1 ppm	Int J Environ Res Public Health. 2016 Jun 28;13(7):642. doi: 10.3390/ijerph13070642.	Global
Cardiac Arrhythmia mortality	NO2	4	1.026	(1.003-1.050)						10 ppb	Int J Environ Res Public Health. 2016 Jun 28;13(7):642. doi: 10.3390/ijerph13070642.	Global
Cardiac Arrhythmia mortality	O3	3	0.997	(0.975-1.018)						10 ppb	Int J Environ Res Public Health. 2016 Jun 28;13(7):642. doi: 10.3390/ijerph13070642.	Global
Cardiac Arrhythmia mortality	PM10	2	1.009	(0.994-1.024)						10 µg/m3	Int J Environ Res Public Health. 2016 Jun 28;13(7):642. doi: 10.3390/ijerph13070642.	Global
Cardiac Arrhythmia mortality	PM2.5	3	1.027	(0.987-1.068)						10 µg/m3	Int J Environ Res Public Health. 2016 Jun 28;13(7):642. doi: 10.3390/ijerph13070642.	Global
Cardiac Arrhythmia mortality	SO2	3	1.028	(0.988-1.069)						10 ppb	Int J Environ Res Public Health. 2016 Jun 28;13(7):642. doi: 10.3390/ijerph13070642.	Global
Cardiac disease mortality	NO2	4	1.0177		23					10 µg/m3	BMC Public Health. 2013 Apr 18;13:360. doi: 10.1186/1471-2458-13-360.	China
Cardiac disease mortality	O3	4	1.0026		31					10 µg/m3	BMC Public Health. 2013 Apr 18;13:360. doi: 10.1186/1471-2458-13-360.	China
Cardiac disease mortality	PM10	4	1.0062		15					10 µg/m3	BMC Public Health. 2013 Apr 18;13:360. doi: 10.1186/1471-2458-13-360.	China
Cardiac disease mortality	SO2	4	1.0182		41					10 µg/m3	BMC Public Health. 2013 Apr 18;13:360. doi: 10.1186/1471-2458-13-360.	China
Cerebrovascular mortality	NO2	5	1.0147		39					10 µg/m3	BMC Public Health. 2013 Apr 18;13:360. doi: 10.1186/1471-2458-13-360.	China
Cerebrovascular mortality	O3	5	1.0057		29					10 µg/m3	BMC Public Health. 2013 Apr 18;13:360. doi: 10.1186/1471-2458-13-360.	China
Cerebrovascular mortality	PM10	5	1.0057		18					10 µg/m3	BMC Public Health. 2013 Apr 18;13:360. doi: 10.1186/1471-2458-13-360.	China
Cerebrovascular mortality	SO2	5	1.0079		26					10 µg/m3	BMC Public Health. 2013 Apr 18;13:360. doi: 10.1186/1471-2458-13-360.	China
Cerebrovascular mortality	PM10	20	1.0044	(1.0022-1.0066)						10 µg/m3	Environ Int. 2020 Sep;142:105876. doi: 10.1016/j.envint.2020.105876. Epub 2020 Jun 23.	Global
Cerebrovascular mortality	PM10	11	1.005	(1.003-1.007)						10 µg/m3	J Am Heart Assoc. 2014 Aug 7;3(4):e000983. doi: 10.1161/JAHA.114.000983.	Global
Cerebrovascular mortality	PM2.5	7	1.0072	(1.0012-1.0132)						10 µg/m3	Environ Int. 2020 Sep;142:105876. doi: 10.1016/j.envint.2020.105876. Epub 2020 Jun 23.	Global
Cerebrovascular mortality	PM2.5	5	1.014	(1.009-1.019)						10 µg/m3	J Am Heart Assoc. 2014 Aug 7;3(4):e000983. doi: 10.1161/JAHA.114.000983.	Global
CVD mortality*	BC	8	1.006	(1.003-1.009)						10 µg/m3	Environ Pollut. 2023 May 1;324:121086. doi: 10.1016/j.envpol.2023.121086. Epub 2023 Jan 14.	Global
CVD mortality*	NO2	15	1.0162		22					10 µg/m3	BMC Public Health. 2013 Apr 18;13:360. doi: 10.1186/1471-2458-13-360.	China
CVD mortality	NO2	10						1.07	(0.43-1.72)	10 µg/m3	BMJ Open. 2016 Jul 21;6(7):e010751. doi: 10.1136/bmjopen-2015-010751.	Global
CVD mortality*	O3	15	1.0051		13					10 µg/m3	BMC Public Health. 2013 Apr 18;13:360. doi: 10.1186/1471-2458-13-360.	China
CVD mortality*	PM10	44	1.006	(1.0044-1.0077)						10 µg/m3	Environ Int. 2020 Sep;142:105876. doi: 10.1016/j.envint.2020.105876. Epub 2020 Jun 23.	Global
CVD mortality	PM10	15	1.0049		7					10 µg/m3	BMC Public Health. 2013 Apr 18;13:360. doi: 10.1186/1471-2458-13-360.	China
CVD mortality	PM10	4						0.48	(0.18-0.78)	10 µg/m3	BMJ Open. 2016 Jul 21;6(7):e010751. doi: 10.1136/bmjopen-2015-010751.	Global
CVD mortality*	PM2.5	28	1.0092	(1.0061-1.0123)						10 µg/m3	Environ Int. 2020 Sep;142:105876. doi: 10.1016/j.envint.2020.105876. Epub 2020 Jun 23.	Global
CVD mortality*	SO2	15	1.0072		17					10 µg/m3	BMC Public Health. 2013 Apr 18;13:360. doi: 10.1186/1471-2458-13-360.	China
Diastolic blood pressure	PM10	6				1.28	(1.02-1.60)			10 µg/m3	Blood Press. 2016 Jun;25(3):169-76. doi: 10.3109/08037051.2015.1111019. Epub 2015 Dec 4.	Global
Diastolic blood pressure	PM2.5	11				1.12	(0.74-1.69)			10 µg/m3	Blood Press. 2016 Jun;25(3):169-76. doi: 10.3109/08037051.2015.1111019. Epub 2015 Dec 4.	Global
Heart failure	CO	20	1.0348	(1.0233-1.048)						1 mg/m3	Front Public Health. 2023 Jan 23;10:948765. doi: 10.3389/fpubh.2022.948765. eCollection 2022.	Global
Heart failure	CO	12	1.035	(1.025-1.045)						1 ppm	Lancet. 2013 Sep 21;382(9897):1039-48. doi: 10.1016/S0140-6736(13)60898-3. Epub 2013 Jul 10.	Global
Heart failure*	CO	41	1.032	(1.023-1.042)						1 ppm	Environ Health Perspect. 2023 Jul;131(7):76001. doi: 10.1289/EHP11506. Epub 2023 Jul 3.	Global
Heart failure*	NO2	44	1.038	(1.019-1.057)						10ppb	Environ Health Perspect. 2023 Jul;131(7):76001. doi: 10.1289/EHP11506. Epub 2023 Jul 3.	Global
Heart failure	NO2	19	1.0207	(1.0138-1.0277)						10ppb	Front Public Health. 2023 Jan 23;10:948765. doi: 10.3389/fpubh.2022.948765. eCollection 2022.	Global
Heart failure	NO2	8				1.016	(1.005-1.026)			10 µg/m3	Sci Total Environ. 2023 May 10;872:162191. doi: 10.1016/j.scitotenv.2023.162191. Epub 2023 Feb 11.	Global
Heart failure	NO2	10	1.017	(1.012-1.022)						10 ppb	Lancet. 2013 Sep 21;382(9897):1039-48. doi: 10.1016/S0140-6736(13)60898-3. Epub 2013 Jul 10.	Global
Heart failure	O3	19	1.0095	(1.0024-1.0166)						10ppb	Front Public Health. 2023 Jan 23;10:948765. doi: 10.3389/fpubh.2022.948765. eCollection 2022.	Global
Heart failure	O3	40	1.01	(0.998-1.021)						10ppb	Environ Health Perspect. 2023 Jul;131(7):76001. doi: 10.1289/EHP11506. Epub 2023 Jul 3.	Global
Heart failure*	PM10	49	1.016	(1.011-1.020)						10 µg/m3	Environ Health Perspect. 2023 Jul;131(7):76001. doi: 10.1289/EHP11506. Epub 2023 Jul 3.	Global
Heart failure	PM10	27	1.013	(1.0102-1.0157)						10 µg/m3	Front Public Health. 2023 Jan 23;10:948765. doi: 10.3389/fpubh.2022.948765. eCollection 2022.	Global
Heart failure	PM10	10				1.012	(1.007-1.017)			10 µg/m3	Sci Total Environ. 2023 May 10;872:162191. doi: 10.1016/j.scitotenv.2023.162191. Epub 2023 Feb 11.	Global
Heart failure*	PM2.5	65	1.018	(1.011-1.025)						10 µg/m3	Environ Health Perspect. 2023 Jul;131(7):76001. doi: 10.1289/EHP11506. Epub 2023 Jul 3.	Global

Cardiovascular condition	Pollutant	Study size	Relative Risk	Relative Risk 95% CI	Standard error in 10-	Odds Ratio	Odds Ratio 95% CI	Percentage increase	Percentage increase 95% CI	Increase in pollutant	Study	Coverage
Heart failure	PM2.5	28	1.0129	(1.0093-1.0165)						10 µg/m3	Front Public Health. 2023 Jan 23;10:948765. doi: 10.3389/fpubh.2022.948765. eCollection 2022.	Global
Heart failure	PM2.5	13				1.019	(1.008-1.030)			10 µg/m3	Sci Total Environ. 2023 May 10;872:162191. doi: 10.1016/j.scitotenv.2023.162191. Epub 2023 Feb 11.	Global
Heart failure	SO2	18	1.022	(1.0108-1.0335)						1 ppb	Front Public Health. 2023 Jan 23;10:948765. doi: 10.3389/fpubh.2022.948765. eCollection 2022.	Global
Heart failure*	SO2	38	1.032	(1.017-1.048)						10ppb	Environ Health Perspect. 2023 Jul;131(7):76001. doi: 10.1289/EHP11506. Epub 2023 Jul 3.	Global
Heart failure mortality/hospitalisation	O3	2	1.005	(0.999-1.011)						10 ppb	Lancet. 2013 Sep 21;382(9897):1039-48. doi: 10.1016/S0140-6736(13)60898-3. Epub 2013 Jul 10.	Global
Heart failure mortality/hospitalisation	PM10	6	1.016	(1.012-1.021)						10 µg/m3	Lancet. 2013 Sep 21;382(9897):1039-48. doi: 10.1016/S0140-6736(13)60898-3. Epub 2013 Jul 10.	Global
Heart failure mortality/hospitalisation	PM2.5	6	1.021	(1.014-1.028)						10 µg/m3	Lancet. 2013 Sep 21;382(9897):1039-48. doi: 10.1016/S0140-6736(13)60898-3. Epub 2013 Jul 10.	Global
Heart failure mortality/hospitalisation	SO2	6	1.024	(1.014-1.034)						10 ppb	Lancet. 2013 Sep 21;382(9897):1039-48. doi: 10.1016/S0140-6736(13)60898-3. Epub 2013 Jul 10.	Global
Hypertension	NO2	3				1.037	(0.994-1.082)			10 µg/m3	Hypertension. 2016 Jul;68(1):62-70. doi: 10.1161/HYPERTENSIONAHA.116.07218. Epub 2016 May 31.	Global
Hypertension	PM10	4				1.024	(1.017-1.030)			10 µg/m3	Hypertension. 2016 Jul;68(1):62-70. doi: 10.1161/HYPERTENSIONAHA.116.07218. Epub 2016 May 31.	Global
Hypertension	PM2.5	3				1.069	(1.003-1.141)			10 µg/m3	Hypertension. 2016 Jul;68(1):62-70. doi: 10.1161/HYPERTENSIONAHA.116.07218. Epub 2016 May 31.	Global
Hypertension	SO2	3				1.046	(1.012-1.081)			10 µg/m3	Hypertension. 2016 Jul;68(1):62-70. doi: 10.1161/HYPERTENSIONAHA.116.07218. Epub 2016 May 31.	Global
IHD morbidity	NO2	34				1.074	(1.052-1.097)			10ppb	Environ Health. 2020 May 1;19(1):47. doi: 10.1186/s12940-020-00601-1.	Global
IHD morbidity	NO2	41	1.022	(1.016-1.029)						10ppb	Environ Health. 2020 May 1;19(1):47. doi: 10.1186/s12940-020-00601-1.	Global
IHD mortality	NO2	2	1.013		118					10 µg/m3	BMC Public Health. 2013 Apr 18;13:360. doi: 10.1186/1471-2458-13-360.	China
IHD mortality	PM10	2	0.9963		52					10 µg/m3	BMC Public Health. 2013 Apr 18;13:360. doi: 10.1186/1471-2458-13-360.	China
IHD mortality	PM2.5	3						3.36	(0.68-6.10)	10 µg/m3	Thorax. 2014 Jul;69(7):660-5. doi: 10.1136/thoraxjnl-2013-204492. Epub 2014 Apr 4.	Global
IHD mortality	SO2	2	1.028		82					10 µg/m3	BMC Public Health. 2013 Apr 18;13:360. doi: 10.1186/1471-2458-13-360.	China
MI hospitalization	PM2.5	16	1.024	(1.007-1.041)						10 µg/m3	Environ Sci Pollut Res Int. 2016 Apr;23(7):6139-48. doi: 10.1007/s11356-016-6186-3. Epub 2016 Feb 5.	Global
MI mortality	PM10	4	1.008	(1.004-1.012)						10 µg/m3	Environ Sci Pollut Res Int. 2016 Apr;23(7):6139-48. doi: 10.1007/s11356-016-6186-3. Epub 2016 Feb 5.	Global
MI mortality	PM2.5	4	1.012	(1.010-1.015)						10 µg/m3	Environ Sci Pollut Res Int. 2016 Apr;23(7):6139-48. doi: 10.1007/s11356-016-6186-3. Epub 2016 Feb 5.	Global
Myocardial infarction risk*	CO	20	1.048	(1.026-1.070)						1 mg/m3	JAMA. 2012 Feb 15;307(7):713-21. doi: 10.1001/jama.2012.126.	Global
Myocardial infarction risk*	NO2	21	1.011	(1.006-1.016)						10 µg/m3	JAMA. 2012 Feb 15;307(7):713-21. doi: 10.1001/jama.2012.126.	Global
Myocardial infarction risk	O3	19	1.003	(0.997-1.010)						10 µg/m3	JAMA. 2012 Feb 15;307(7):713-21. doi: 10.1001/jama.2012.126.	Global
Myocardial infarction risk*	PM10	17	1.006	(1.002-1.009)						10 µg/m3	JAMA. 2012 Feb 15;307(7):713-21. doi: 10.1001/jama.2012.126.	Global
Myocardial infarction risk*	PM2.5	13	1.025	(1.015-1.036)						10 µg/m3	JAMA. 2012 Feb 15;307(7):713-21. doi: 10.1001/jama.2012.126.	Global
Myocardial infarction risk*	SO2	14	1.01	(1.003-1.017)						10 µg/m3	JAMA. 2012 Feb 15;307(7):713-21. doi: 10.1001/jama.2012.126.	Global
Stroke incidence	CO	5				0.999	(0.997-1.001)			10 µg/m3	Environ Health Prev Med. 2021 Jan 26;26(1):15. doi: 10.1186/s12199-021-00937-1.	Global
Stroke incidence	NO2	7				1.002	(1.000-1.003)			10 µg/m3	Environ Health Prev Med. 2021 Jan 26;26(1):15. doi: 10.1186/s12199-021-00937-1.	Global
Stroke incidence	O3	10				0.999	(0.999-1.000)			10 µg/m3	Environ Health Prev Med. 2021 Jan 26;26(1):15. doi: 10.1186/s12199-021-00937-1.	Global
Stroke incidence	PM10	13				1.017	(0.981-1.055)			10 µg/m3	Environ Health Prev Med. 2021 Jan 26;26(1):15. doi: 10.1186/s12199-021-00937-1.	Global
Stroke incidence	PM2.5	18				1.048	(1.020-1.076)			10 µg/m3	Environ Health Prev Med. 2021 Jan 26;26(1):15. doi: 10.1186/s12199-021-00937-1.	Global
Stroke incidence	SO2	4				1.002	(1.000-1.003)			10 µg/m3	Environ Health Prev Med. 2021 Jan 26;26(1):15. doi: 10.1186/s12199-021-00937-1.	Global
Stroke mortality	CO	5				1.045	(0.980-1.115)			10 µg/m3	Environ Health Prev Med. 2021 Jan 26;26(1):15. doi: 10.1186/s12199-021-00937-1.	Global
Stroke mortality	CO	37	1.054	(0.999-1.108)						1 ppm	BMJ. 2015 Mar 24;350:h1295. doi: 10.1136/bmj.h1295.	Global
Stroke mortality*	NO2	70	1.016	(1.007-1.023)						10 ppb	BMJ. 2015 Mar 24;350:h1295. doi: 10.1136/bmj.h1295.	Global
Stroke mortality	NO2	5						1.76	(0.68-2.85)	10 µg/m3	BMJ Open. 2016 Jul 21;6(7):e010751. doi: 10.1136/bmjopen-2015-010751.	Global
Stroke mortality	NO2	10				1.009	(1.003-1.016)			10 µg/m3	Environ Health Prev Med. 2021 Jan 26;26(1):15. doi: 10.1186/s12199-021-00937-1.	Global
Stroke mortality	O3	6				1.005	(0.999-1.010)			10 µg/m3	Environ Health Prev Med. 2021 Jan 26;26(1):15. doi: 10.1186/s12199-021-00937-1.	Global
Stroke mortality*	O3	53	1.004	(1.001-1.006)						10 ppb	BMJ. 2015 Mar 24;350:h1295. doi: 10.1136/bmj.h1295.	Global
Stroke mortality*	PM10	78	1.003	(1.002-1.004)						10 µg/m3	BMJ. 2015 Mar 24;350:h1295. doi: 10.1136/bmj.h1295.	Global
Stroke mortality	PM10	10				1.006	(1.003-1.010)			10 µg/m3	Environ Health Prev Med. 2021 Jan 26;26(1):15. doi: 10.1186/s12199-021-00937-1.	Global
Stroke mortality	PM2.5	12				1.008	(1.005-1.012)			10 µg/m3	Environ Health Prev Med. 2021 Jan 26;26(1):15. doi: 10.1186/s12199-021-00937-1.	Global
Stroke mortality	PM2.5	3						1.85	(0.74-2.97)	10 µg/m3	Thorax. 2014 Jul;69(7):660-5. doi: 10.1136/thoraxjnl-2013-204492. Epub 2014 Apr 4.	Global
Stroke mortality*	PM2.5	41	1.012	(1.011-1.012)						10 µg/m3	BMJ. 2015 Mar 24;350:h1295. doi: 10.1136/bmj.h1295.	Global
Stroke mortality	SO2	6				1.006	(1.005-1.008)			10 µg/m3	Environ Health Prev Med. 2021 Jan 26;26(1):15. doi: 10.1186/s12199-021-00937-1.	Global
Stroke mortality*	SO2	52	1.022	(1.014-1.031)						10 ppb	BMJ. 2015 Mar 24;350:h1295. doi: 10.1136/bmj.h1295.	Global
Systolic blood pressure	PM10	6				1.25	(0.99-1.58)			10 µg/m3	Blood Press. 2016 Jun;25(3):169-76. doi: 10.3109/08037051.2015.1111019. Epub 2015 Dec 4.	Global
Systolic blood pressure	PM2.5	11				1.38	(0.84-2.28)			10 µg/m3	Blood Press. 2016 Jun;25(3):169-76. doi: 10.3109/08037051.2015.1111019. Epub 2015 Dec 4.	Global

Appendix Table 2: Full set of meta-analysis data, long-term effect of air pollution

Cardiovascular condition	Pollutant	Study size	Relative Risk	Relative Risk 95% CI	Odds Ratio	Odds Ratio 95% CI	Hazard Ratio	Hazard Ratio 95% CI	Percentage increase	Percentage increase 95% CI	Increase in pollutant	Study	Coverage
Acute coronary events	NOx	11					1.01	(0.98 -1.05)			20 μg/m3	BMJ. 2014 Jan 21;348:f7412. doi: 10.1136/bmj.f7412.	Europe
Acute coronary events	NO2	11					1.03	(0.97 -1.08)			10 μg/m3	BMJ. 2014 Jan 21;348:f7412. doi: 10.1136/bmj.f7412.	Europe
Acute coronary events	PM10	11					1.12	(1.01 -1.25)			10 μg/m3	BMJ. 2014 Jan 21;348:f7412. doi: 10.1136/bmj.f7412.	Europe
Acute coronary events	PM2.5	11					1.13	(0.98 -1.30)			5 μg/m3	BMJ. 2014 Jan 21;348:f7412. doi: 10.1136/bmj.f7412.	Europe
Atrial Fibrillation incidence	CO	2					1.017	(1.013-1.022)			1000 ppb	Int Heart J. 2021 Mar 30;62(2):290-297. doi: 10.1536/ihj.20-523. Epub 2021 Mar 6.	Global
Atrial Fibrillation incidence	NO2	4					1.017	(1.001-1.033)			10 ppb	Int Heart J. 2021 Mar 30;62(2):290-297. doi: 10.1536/ihj.20-523. Epub 2021 Mar 6.	Global
Atrial Fibrillation incidence	NO2	2					1.01	(1.01-1.02)			10 μg/m3	J Evid Based Med. 2020 May;13(2):102-115. doi: 10.1111/jebm.12380. Epub 2020 Mar 13.	Global
Atrial Fibrillation incidence	O3	3					1.007	(0.927-1.094)			10 ppb	Int Heart J. 2021 Mar 30;62(2):290-297. doi: 10.1536/ihj.20-523. Epub 2021 Mar 6.	Global
Atrial Fibrillation incidence	PM10	4					1.034	(1.032-1.035)			10 μg/m3	Int Heart J. 2021 Mar 30;62(2):290-297. doi: 10.1536/ihj.20-523. Epub 2021 Mar 6.	Global
Atrial Fibrillation incidence	PM10	4					0.91	(0.74-1.12)			10 μg/m3	J Evid Based Med. 2020 May;13(2):102-115. doi: 10.1111/jebm.12380. Epub 2020 Mar 13.	Global
Atrial Fibrillation incidence	PM2.5	4					0.93	(0.68-1.27)			5 and 10 μg/m3	J Evid Based Med. 2020 May;13(2):102-115. doi: 10.1111/jebm.12380. Epub 2020 Mar 13.	Global
Atrial Fibrillation incidence	SO2	2					1.005	(1.004-1.007)			10 ppb	Int Heart J. 2021 Mar 30;62(2):290-297. doi: 10.1536/ihj.20-523. Epub 2021 Mar 6.	Global
Atrial Fibrillation incidence*	PM2.5	6					1.116	(1.031-1.207)			10 μg/m3	Int Heart J. 2021 Mar 30;62(2):290,297. doi: 10.1536/ihj.20,523. Epub 2021 Mar 6.	Global
Atrial fibrillation prevalence	NO2	3			1.02	(1.00-1.04)					10 ppb	Ecotoxicol Environ Saf. 2021 Jan 15;208:111508. doi: 10.1016/j.ecoenv.2020.111508. Epub 2020 Nov 1.	Global
Atrial fibrillation prevalence	PM10	3			1.03	(1.03-1.04)					10 μg/m3	Ecotoxicol Environ Saf. 2021 Jan 15;208:111508. doi: 10.1016/j.ecoenv.2020.111508. Epub 2020 Nov 1.	Global
Atrial fibrillation prevalence	PM2.5	4			1.07	(1.04-1.10)					10 μg/m3	Ecotoxicol Environ Saf. 2021 Jan 15;208:111508. doi: 10.1016/j.ecoenv.2020.111508. Epub 2020 Nov 1.	Global
Cerebrovascular mortality	BC	3	1.082	(0.920-1.273)							10 μg/m3	Environ Pollut. 2023 May 1;324:121086. doi: 10.1016/j.envpol.2023.121086. Epub 2023 Jan 14.	Global
Cerebrovascular mortality	NO2	17					1.167	(0.936-1.456)			10 ppb	PLoS One. 2021 Feb 4;16(2):e0246451. doi: 10.1371/journal.pone.0246451. eCollection 2021.	Global
Cerebrovascular mortality	PM2.5	n=3740					1.128	(1.048-1.214)			5 μg/m3	Res Rep Health Eff Inst. 2021 Sep;2021(208):1-127.	Europe
Cerebrovascular mortality	BC	n=3740					1.075	(1.016-1.138)			0.5x10−5/m	Res Rep Health Eff Inst. 2021 Sep;2021(208):1-127.	Europe
Cerebrovascular mortality	NO2	n=3740					1.068	(1.011-1.129)			10 μg/m3	Res Rep Health Eff Inst. 2021 Sep;2021(208):1-127.	Europe
Cerebrovascular mortality	O3	n=3740					0.882	(0.817-0.953)			10 μg/m3	Res Rep Health Eff Inst. 2021 Sep;2021(208):1-127.	Europe
Cerebrovascular mortality	PM2.5	17					1.24	(1.13-1.36)			10 μg/m3	J Am Heart Assoc. 2021 Jan 5;10(1):e016890. doi: 10.1161/JAHA.120.016890. Epub 2020 Dec 31.	Global
Coronary heart disease incidence	BC	n=137148					1.02	(0.99-1.06)			10−5/m	Lancet Planet Health. 2021 Sep;5(9):e620-e632. doi: 10.1016/S2542-5196(21)00195-9.	Europe
Coronary heart disease incidence	NO2	n=137148					1.04	(1.01-1.07)			10 μg/m3	Lancet Planet Health. 2021 Sep;5(9):e620-e632. doi: 10.1016/S2542-5196(21)00195-9.	Europe
Coronary heart disease incidence	O3	n=137148					0.94	(0.90-0.98)			10 μg/m3	Lancet Planet Health. 2021 Sep;5(9):e620-e632. doi: 10.1016/S2542-5196(21)00195-9.	Europe
Coronary heart disease incidence	PM2.5	n=137148					1.02	(0.95-1.10)			5 μg/m3	Lancet Planet Health. 2021 Sep;5(9):e620-e632. doi: 10.1016/S2542-5196(21)00195-9.	Europe
Coronary heart disease incidence	NO2	6					1.02	(0.95-1.10)			10 μg/m3	J Evid Based Med. 2020 May;13(2):102-115. doi: 10.1111/jebm.12380. Epub 2020 Mar 13.	Global
Coronary heart disease incidence	PM10	9					1.09	(0.98-1.21)			10 μg/m3	J Evid Based Med. 2020 May;13(2):102-115. doi: 10.1111/jebm.12380. Epub 2020 Mar 13.	Global
Coronary heart disease incidence	PM2.5	8					1.04	(1.00-1.09)			5 and 10 μg/m3	J Evid Based Med. 2020 May;13(2):102-115. doi: 10.1111/jebm.12380. Epub 2020 Mar 13.	Global
Coronary heart disease mortality	BC	3	1.209	(0.921-1.588)							10 μg/m3	Environ Pollut. 2023 May 1;324:121086. doi: 10.1016/j.envpol.2023.121086. Epub 2023 Jan 14.	Global
CVD morbidity	NO2	10					1.108	(1.007-1.219)			10 μg/m3	Environ Int. 2023 Aug;178:108060. doi: 10.1016/j.envint.2023.108060. Epub 2023 Jun 23.	China
CVD morbidity	PM2.5	5					1.09	(1.00-1.18)			10 μg/m3	Curr Probl Cardiol. 2023 Jun;48(6):101670. doi: 10.1016/j.cpcardiol.2023.101670. Epub 2023 Feb 23.	Global
CVD morbidity	PM10	7	1.07	(1.01-1.13)							10 μg/m3	Front Public Health. 2023 Mar 28;11:1134341. doi: 10.3389/fpubh.2023.1134341. eCollection 2023.	LMIC
CVD morbidity	PM2.5	10	1.11	(1.05-1.17)							10 μg/m3	Front Public Health. 2023 Mar 28;11:1134341. doi: 10.3389/fpubh.2023.1134341. eCollection 2023.	LMIC
CVD mortality	BC	n=15542					1.085	(1.055-1.116)			0.5x10−5/m	Res Rep Health Eff Inst. 2021 Sep;2021(208):1-127.	Europe
CVD mortality	NO2	29					1.139	(0.997-1.301)			10 ppb	PLoS One. 2021 Feb 4;16(2):e0246451. doi: 10.1371/journal.pone.0246451. eCollection 2021.	Global
CVD mortality	NO2	6					1.14	(0.89-1.47)			10 ppb	Res Rep Health Eff Inst. 2023 May;2016(213):1-53.	Asia
CVD mortality	NO2	n=15542					1.089	(1.060-1.120)			10 μg/m3	Res Rep Health Eff Inst. 2021 Sep;2021(208):1-127.	Europe
CVD mortality	O3	6	1.01	(0.99-1.03)							10 ppb	BMJ Open. 2016 Feb 23;6(2):e009493. doi: 10.1136/bmjopen-2015-009493.	Global
CVD mortality	PM10	5	1.14	(0.99-1.30)							10 μg/m3	Front Public Health. 2023 Mar 28;11:1134341. doi: 10.3389/fpubh.2023.1134341. eCollection 2023.	Asia
CVD mortality	PM10	5					1.02	(0.89-1.16)			10 μg/m3	Environ Pollut. 2018 Nov;242(Pt B):1299-1307. doi: 10.1016/j.envpol.2018.07.041. Epub 2018 Jul 28.	Global
CVD mortality	PM2.5	18					1.1	(1.07-1.12)			5 and 10 μg/m3	J Evid Based Med. 2020 May;13(2):102-115. doi: 10.1111/jebm.12380. Epub 2020 Mar 13.	Global
CVD mortality	PM2.5	11	1.1	(1.06-1.14)							10 μg/m3	Front Public Health. 2023 Mar 28;11:1134341. doi: 10.3389/fpubh.2023.1134341. eCollection 2023.	LMIC
CVD mortality	PM2.5	14					1.12	(1.07-1.18)			10 μg/m3	Curr Probl Cardiol. 2023 Jun;48(6):101670. doi: 10.1016/j.cpcardiol.2023.101670. Epub 2023 Feb 23.	Global
CVD mortality	PM2.5	6					1.05	(0.99-1.12)			5 μg/m3	Res Rep Health Eff Inst. 2023 May;2016(213):1-53.	Asia
CVD mortality	PM2.5	11					1.12	(1.08-1-16)			10 μg/m3	Environ Pollut. 2018 Nov;242(Pt B):1299-1307. doi: 10.1016/j.envpol.2018.07.041. Epub 2018 Jul 28.	Global
CVD mortality	PM2.5	n=15542					1.135	(1.095-1.176)			5 μg/m3	Res Rep Health Eff Inst. 2021 Sep;2021(208):1-127.	Europe
CVD mortality	BC	13	1.093	(0.955-1.250)							10 μg/m3	Environ Pollut. 2023 May 1;324:121086. doi: 10.1016/j.envpol.2023.121086. Epub 2023 Jan 14.	Global
CVD mortality*	NO2	13					1.17	(1.10-1.25)			10 μg/m3	J Evid Based Med. 2020 May;13(2):102-115. doi: 10.1111/jebm.12380. Epub 2020 Mar 13.	Global
CVD mortality*	O3	n=15542					0.887	(0.854-0.922)			10 μg/m3	Res Rep Health Eff Inst. 2021 Sep;2021(208):1-127.	Europe
CVD mortality*	PM10	11					1.17	(1.04-1.30)			10 μg/m3	J Evid Based Med. 2020 May;13(2):102-115. doi: 10.1111/jebm.12380. Epub 2020 Mar 13.	Global
CVD mortality*	PM2.5	28					1.14	(1.08-1.21)			10 μg/m3	J Am Heart Assoc. 2021 Jan 5;10(1):e016890. doi: 10.1161/JAHA.120.016890. Epub 2020 Dec 31.	Global
Diastolic blood pressure	PM10	1			1.18	(1.04-1.34)					10 μg/m3	Blood Press. 2016 Jun;25(3):169-76. doi: 10.3109/08037051.2015.1111019. Epub 2015 Dec 4.	Global
Diastolic blood pressure	PM2.5	2			4.52	(0.23-89.77)					10 μg/m3	Blood Press. 2016 Jun;25(3):169-76. doi: 10.3109/08037051.2015.1111019. Epub 2015 Dec 4.	Global
Heart failure incidence	NO2	3					1.42	(0.93-2.18)			10 μg/m3	J Evid Based Med. 2020 May;13(2):102-115. doi: 10.1111/jebm.12380. Epub 2020 Mar 13.	Global
Heart failure incidence	PM10	5					1.25	(1.04-1.50)			10 μg/m3	J Evid Based Med. 2020 May;13(2):102-115. doi: 10.1111/jebm.12380. Epub 2020 Mar 13.	Global
Heart failure incidence	PM2.5	4					1.07	(0.72-1.60)			5 and 10 μg/m3	J Evid Based Med. 2020 May;13(2):102-115. doi: 10.1111/jebm.12380. Epub 2020 Mar 13.	Global
Heart failure incidence-mortality	CO	3	2.265	(0.474-10.832)							1 ppm	Environ Health Perspect. 2023 Jul;131(7):76001. doi: 10.1289/EHP11506. Epub 2023 Jul 3.	Global
Heart failure incidence-mortality	NO2	8			1.072	(1.028-1.118)					10 μg/m3	Sci Total Environ. 2023 May 10;872:162191. doi: 10.1016/j.scitotenv.2023.162191. Epub 2023 Feb 11.	Global
Heart failure incidence-mortality	O3	5	1.011	(0.860-1.187)							10ppb	Environ Health Perspect. 2023 Jul;131(7):76001. doi: 10.1289/EHP11506. Epub 2023 Jul 3.	Global
Heart failure incidence-mortality	PM10	8			1.19	(1.045-1.356)					10 μg/m3	Sci Total Environ. 2023 May 10;872:162191. doi: 10.1016/j.scitotenv.2023.162191. Epub 2023 Feb 11.	Global
Heart failure incidence-mortality	PM2.5	13			1.196	(1.079-1.326)					10 μg/m3	Sci Total Environ. 2023 May 10;872:162191. doi: 10.1016/j.scitotenv.2023.162191. Epub 2023 Feb 11.	Global
Heart failure incidence-mortality	SO2	2	3.929	(0.282-54.798)							10ppb	Environ Health Perspect. 2023 Jul;131(7):76001. doi: 10.1289/EHP11506. Epub 2023 Jul 3.	Global
Heart failure incidence-mortality*	NO2	10	1.204	(1.069-1.356)							10ppb	Environ Health Perspect. 2023 Jul;131(7):76001. doi: 10.1289/EHP11506. Epub 2023 Jul 3.	Global
Heart failure incidence-mortality*	PM10	8	1.212	(1.010-1.454)							10 μg/m3	Environ Health Perspect. 2023 Jul;131(7):76001. doi: 10.1289/EHP11506. Epub 2023 Jul 3.	Global
Heart failure incidence-mortality*	PM2.5	15	1.748	(1.112-2.747)							10 μg/m3	Environ Health Perspect. 2023 Jul;131(7):76001. doi: 10.1289/EHP11506. Epub 2023 Jul 3.	Global
Hemorrhagic stroke incidence or mort	PM2.5	7					1.1	(1.04-1.16)			was not unified, which ranged from 1.4 μg/m3 to 10 μg/m3.	Environ Sci Pollut Res Int. 2021 May;28(17):20970-20980. doi: 10.1007/s11356-021-13074-7. Epub 2021 Mar 10.	Global
Hypertension incidence	PM2.5	10	1.21	(1.07-1.35)							10 μg/m3	Environ Res. 2022 Mar;204(Pt D):112352. doi: 10.1016/j.envres.2021.112352. Epub 2021 Nov 9.	Global
Hypertension incidence-women	PM2.5	6					1.23	(1.08-1.40)			10 μg/m3	Ecotoxicol Environ Saf. 2021 Jan 15;208:111492. doi: 10.1016/j.ecoenv.2020.111492. Epub 2020 Oct 26.	Global
Hypertension incidence	PM2.5	10					1.07	(1.01-1.14)			5 and 10 μg/m3	J Evid Based Med. 2020 May;13(2):102-115. doi: 10.1111/jebm.12380. Epub 2020 Mar 13.	Global
Hypertension prevalence	NO2	6			1.034	(1.005-1.063)					10 μg/m3	Hypertension. 2016 Jul;68(1):62-70. doi: 10.1161/HYPERTENSIONAHA.116.07218. Epub 2016 May 31.	Global
Hypertension prevalence	NOx	4			1.127	(0.933-1.361)					10 μg/m3	Hypertension. 2016 Jul;68(1):62,70. doi: 10.1161/HYPERTENSIONAHA.116.07218. Epub 2016 May 31.	Global
Hypertension prevalence	PM10	3			1.054	(1.036-1.072)					10 μg/m3	Hypertension. 2016 Jul;68(1):62-70. doi: 10.1161/HYPERTENSIONAHA.116.07218. Epub 2016 May 31.	Global
Hypertension prevalence	PM10	11			1.11	(1.07-1.16)					10 μg/m3	Int J Environ Health Res. 2023 Mar;33(3):268-283. doi: 10.1080/09603123.2021.2022106. Epub 2022 Jan 4.	Global
Hypertension prevalence	PM2.5	5			1.065	(0.985-1.152)					10 μg/m3	Hypertension. 2016 Jul;68(1):62-70. doi: 10.1161/HYPERTENSIONAHA.116.07218. Epub 2016 May 31.	Global
Hypertension prevalence	PM2.5	26			1.15	(1.10-1.20)					10 μg/m3	Int J Environ Health Res. 2023 Mar;33(3):268-283. doi: 10.1080/09603123.2021.2022106. Epub 2022 Jan 4.	Global
Hypertension prevalence	PM2.5	18			1.06	(1.03-1.09)					10 μg/m3	Environ Res. 2022 Mar;204(Pt D):112352. doi: 10.1016/j.envres.2021.112352. Epub 2021 Nov 9.	Global
Hypertension prevalence	CO	1			1.13	(1.00-1.28)					10 μg/m3	Sci Total Environ. 2021 Nov 20;796:148620. doi: 10.1016/j.scitotenv.2021.148620. Epub 2021 Jul 7.	Global
Hypertension prevalence	NO2	27			1.01	(1.00-1.03)					10 μg/m3	Sci Total Environ. 2021 Nov 20;796:148620. doi: 10.1016/j.scitotenv.2021.148620. Epub 2021 Jul 7.	Global

Cardiovascular condition	Pollutant	Study size	Relative Risk	Relative Risk 95% CI	Odds Ratio	Odds Ratio 95% CI	Hazard Ratio	Hazard Ratio 95% CI	Percentage increase	Percentage increase 95% CI	Increase in pollutant	Study	Coverage
Hypertension prevalence	NOx	17			1.01	(0.98-1.03)					10 µg/m3	Sci Total Environ. 2021 Nov 20;796:148620. doi: 10.1016/j.scitotenv.2021.148620. Epub 2021 Jul 7.	Global
Hypertension prevalence	O3	3			0.96	(0.85-1.09)					10 µg/m3	Sci Total Environ. 2021 Nov 20;796:148620. doi: 10.1016/j.scitotenv.2021.148620. Epub 2021 Jul 7.	Global
Hypertension prevalence	PM10	22			1.04	(1.02-1.06)					10 µg/m3	Sci Total Environ. 2021 Nov 20;796:148620. doi: 10.1016/j.scitotenv.2021.148620. Epub 2021 Jul 7.	Global
Hypertension prevalence	PM2.5	37			1.1	(1.07-1.14)					10 µg/m3	Sci Total Environ. 2021 Nov 20;796:148620. doi: 10.1016/j.scitotenv.2021.148620. Epub 2021 Jul 7.	Global
Hypertension prevalence	SO2	3			1.21	(1.08-1.36)					10 µg/m3	Sci Total Environ. 2021 Nov 20;796:148620. doi: 10.1016/j.scitotenv.2021.148620. Epub 2021 Jul 7.	Global
Hypertension prevalence-Child	CO	1			1.009	(1.007-1.011)					10 µg/m3	Sci Total Environ. 2021 Nov 20;796:148620. doi: 10.1016/j.scitotenv.2021.148620. Epub 2021 Jul 7.	Global
Hypertension prevalence-Child	NO2	1			1.02	(1.00-1.03)					10 µg/m3	Sci Total Environ. 2021 Nov 20;796:148620. doi: 10.1016/j.scitotenv.2021.148620. Epub 2021 Jul 7.	Global
Hypertension prevalence-Child	O3	2			1.26	(0.81-1.09)					10 µg/m3	Sci Total Environ. 2021 Nov 20;796:148620. doi: 10.1016/j.scitotenv.2021.148620. Epub 2021 Jul 7.	Global
Hypertension prevalence-Child	PM10	2			1.15	(1.01-1.32)					10 µg/m3	Sci Total Environ. 2021 Nov 20;796:148620. doi: 10.1016/j.scitotenv.2021.148620. Epub 2021 Jul 7.	Global
Hypertension prevalence-Child	PM2.5	2			2.82	(0.51-15.68)					10 µg/m3	Sci Total Environ. 2021 Nov 20;796:148620. doi: 10.1016/j.scitotenv.2021.148620. Epub 2021 Jul 7.	Global
Hypertension prevalence-Child	SO2	2			8.57	(0.13-575.58)					10 µg/m3	Sci Total Environ. 2021 Nov 20;796:148620. doi: 10.1016/j.scitotenv.2021.148620. Epub 2021 Jul 7.	Global
Hypertension prevalence-women	PM2.5	4			1.07	(1.00-1.14)					10 µg/m3	Ecotoxicol Environ Saf. 2021 Jan 15;208:111492. doi: 10.1016/j.ecoenv.2020.111492. Epub 2020 Oct 26.	Global
IHD (women to men ratio-RRR)	PM2.5	9	1.05	(1.02-1.08)							10 µg/m3	Front Public Health. 2022 Feb 2;10:802167. doi: 10.3389/fpubh.2022.802167. eCollection 2022.	Global
IHD incidence	PM2.5	4	1.07	(0.99-1.17)							10 µg/m3	Perfusion. 2024 Jan;39(1):210-222. doi: 10.1177/02676591221131485. Epub 2022 Nov 7.	Global
IHD mortality	BC	n=7265					1.078	(1.033-1.125)			0.5x10−5/m	Res Rep Health Eff Inst. 2021 Sep;2021(208):1-127.	Europe
IHD mortality	NO2	6					1.05	(1.03-1.08)			10 µg/m3	J Evid Based Med. 2020 May;13(2):102-115. doi: 10.1111/jebm.12380. Epub 2020 Mar 13.	Global
IHD mortality	NO2	n=7265					1.098	(1.053-1.145)			10 µg/m3	Res Rep Health Eff Inst. 2021 Sep;2021(208):1-127.	Europe
IHD mortality	O3	4	1.02	(1.00.1.04)							10 ppb	BMJ Open. 2016 Feb 23;6(2):e009493. doi: 10.1136/bmjopen-2015-009493.	Global
IHD mortality	PM10	5					1.03	(1.01-1.05)			10 µg/m3	J Evid Based Med. 2020 May;13(2):102-115. doi: 10.1111/jebm.12380. Epub 2020 Mar 13.	Global
IHD mortality	PM2.5	15					1.11	(1.07-1.16)			5 and 10 µg/m3	J Evid Based Med. 2020 May;13(2):102-115. doi: 10.1111/jebm.12380. Epub 2020 Mar 13.	Global
IHD mortality	PM2.5	23					1.23	(1.15-1.31)			10 µg/m3	J Am Heart Assoc. 2021 Jan 5;10(1):e016890. doi: 10.1161/JAHA.120.016890. Epub 2020 Dec 31.	Global
IHD mortality	PM2.5	n=7265					1.111	(1.056-1.169)			5 µg/m3	Res Rep Health Eff Inst. 2021 Sep;2021(208):1-127.	Europe
IHD mortality	PM2.5	22	1.16	(1.10-1.21)							10 µg/m3	Environ Int. 2020 Oct;143:105974. doi: 10.1016/j.envint.2020.105974. Epub 2020 Jul 20.	Global
IHD mortality*	BC	8	1.149	(1.024-1.291)							10 µg/m3	Environ Pollut. 2023 May 1;324:121086. doi: 10.1016/j.envpol.2023.121086. Epub 2023 Jan 14.	Global
IHD mortality*	NO2	19					1.128	(1.076-1.182)			10 ppb	PLoS One. 2021 Feb 4;16(2):e0246451. doi: 10.1371/journal.pone.0246451. eCollection 2021.	Global
IHD mortality*	O3	n=7265					0.87	(0.821-0.921)			10 µg/m3	Res Rep Health Eff Inst. 2021 Sep;2021(208):1-127.	Europe
IHD mortality*	PM10	13	1.06	(1.01-1.10)							10 µg/m3	Environ Int. 2020 Oct;143:105974. doi: 10.1016/j.envint.2020.105974. Epub 2020 Jul 20.	Global
IHD mortality*	PM2.5	24	1.21	(1.15-1.28)							10 µg/m3	Perfusion. 2024 Jan;39(1):210-222. doi: 10.1177/02676591221131485. Epub 2022 Nov 7.	Global
MI incidence	PM2.5	11					1.08	(0.99-1.18)			10 µg/m3	J Am Heart Assoc. 2021 Jan 5;10(1):e016890. doi: 10.1161/JAHA.120.016890. Epub 2020 Dec 31.	Global
MI incidence	PM2.5	8					1.1	(1.02-1.18)			10 µg/m3	Chemosphere. 2021 Mar;267:128903. doi: 10.1016/j.chemosphere.2020.128903. Epub 2020 Nov 7.	Global
Post-MI mortality	PM2.5	4					1.07	(1.04-1.9)			10 µg/m3	Chemosphere. 2021 Mar;267:128903. doi: 10.1016/j.chemosphere.2020.128903. Epub 2020 Nov 7.	Global
Stroke incidence	O3	n=137148					0.96	(0.91-1.01)			10 µg/m3	Lancet Planet Health. 2021 Sep;5(9):e620-e632. doi: 10.1016/S2542-5196(21)00195-9.	Europe
Stroke incidence	PM2.5	14					1.13	(1.11-1.15)			10 µg/m3	J Am Heart Assoc. 2021 Jan 5;10(1):e016890. doi: 10.1161/JAHA.120.016890. Epub 2020 Dec 31.	Global
Stroke incidence	NO2	11					1.13	(1.00-1.28)			10 µg/m3	J Evid Based Med. 2020 May;13(2):102-115. doi: 10.1111/jebm.12380. Epub 2020 Mar 13.	Global
Stroke incidence	PM10	13					1.15	(0.90-1.47)			10 µg/m3	J Evid Based Med. 2020 May;13(2):102-115. doi: 10.1111/jebm.12380. Epub 2020 Mar 13.	Global
Stroke incidence	PM2.5	15					1.13	(1.06-1.19)			5 and 10 µg/m3	J Evid Based Med. 2020 May;13(2):102-115. doi: 10.1111/jebm.12380. Epub 2020 Mar 13.	Global
Stroke incidence	PM2.5	11	1	(0.96-1.04)							10 µg/m3	Front Public Health. 2022 Feb 2;10:802167. doi: 10.3389/fpubh.2022.802167. eCollection 2022.	Global
Stroke incidence	PM10 or conv	20					1.061	(1.018-1.105)			10 µg/m3	Stroke. 2015 Nov;46(11):3058-66. doi: 10.1161/STROKEAHA.115.009913. Epub 2015 Oct 13.	Global
Stroke incidence*	BC	n=137148					1.06	(1.02-1.10)			10−5/m	Lancet Planet Health. 2021 Sep;5(9):e620-e632. doi: 10.1016/S2542-5196(21)00195-9.	Europe
Stroke incidence*	NO2	n=137148					1.08	(1.04-1.12)			10 µg/m3	Lancet Planet Health. 2021 Sep;5(9):e620-e632. doi: 10.1016/S2542-5196(21)00195-9.	Europe
Stroke incidence*	PM2.5	n=137148					1.1	(1.01-1.21)			5 µg/m3	Lancet Planet Health. 2021 Sep;5(9):e620-e632. doi: 10.1016/S2542-5196(21)00195-9.	Europe
Stroke mortality	O3	2							1.01	(0.97-1.05)	10 ppb	BMJ Open. 2016 Feb 23;6(2):e009493. doi: 10.1136/bmjopen-2015-009493.	Global
Stroke mortality	O3	20							1.35	(−0.49-3.22)	10 ppb	Int J Cardiol. 2014 Aug 1;175(2):307-13. doi: 10.1016/j.ijcard.2014.05.044. Epub 2014 May 17.	Global
Stroke mortality	PM10	9							1.01	(0.83-1.21)	10 µg/m3	Environ Int. 2020 Oct;143:105974. doi: 10.1016/j.envint.2020.105974. Epub 2020 Jul 20.	Global
Stroke mortality	PM10 or conv	12	1.08	(0.992-1.177)							10 µg/m3	Stroke. 2015 Nov;46(11):3058-66. doi: 10.1161/STROKEAHA.115.009913. Epub 2015 Oct 13.	Global
Stroke mortality	PM2.5	8							1.34	(0.27-2.42)	10 µg/m3	Int J Cardiol. 2014 Aug 1;175(2):307-13. doi: 10.1016/j.ijcard.2014.05.044. Epub 2014 May 17.	Global
Stroke mortality*	CO	16							7.78	(4.49-11.60)	1 ppm	Int J Cardiol. 2014 Aug 1;175(2):307-13. doi: 10.1016/j.ijcard.2014.05.044. Epub 2014 May 17.	Global
Stroke mortality*	NO2	24							1.5	(0.37-2.63)	10 ppb	Int J Cardiol. 2014 Aug 1;175(2):307-13. doi: 10.1016/j.ijcard.2014.05.044. Epub 2014 May 17.	Global
Stroke mortality*	PM10	21							0.65	(0.54-0.77)	10 µg/m3	Int J Cardiol. 2014 Aug 1;175(2):307-13. doi: 10.1016/j.ijcard.2014.05.044. Epub 2014 May 17.	Global
Stroke mortality*	PM2.5	16	1.11	(1.04-1.18)							10 µg/m3	Environ Int. 2020 Oct;143:105974. doi: 10.1016/j.envint.2020.105974. Epub 2020 Jul 20.	Global
Stroke mortality*	SO2	22							2.45	(1.83-3.07)	10 ppb	Int J Cardiol. 2014 Aug 1;175(2):307-13. doi: 10.1016/j.ijcard.2014.05.044. Epub 2014 May 17.	Global
Systolic blood pressure	PM10	1			1.58	(1.29-1.95)					10 µg/m3	Blood Press. 2016 Jun;25(3):169-76. doi: 10.3109/08037051.2015.1111019. Epub 2015 Dec 4.	Global
Systolic blood pressure	PM2.5	3			4.18	(1.66-10.48)					10 µg/m3	Blood Press. 2016 Jun;25(3):169-76. doi: 10.3109/08037051.2015.1111019. Epub 2015 Dec 4.	Global
